# Synthetic thick filaments: A new avenue for better understanding the myosin super-relaxed state in healthy, diseased, and mavacamten-treated cardiac systems

**DOI:** 10.1074/jbc.RA120.016506

**Published:** 2020-12-03

**Authors:** Sampath K. Gollapudi, Ming Yu, Qing-Fen Gan, Suman Nag

**Affiliations:** Department of Biology, MyoKardia, Inc., Brisbane, California, USA

**Keywords:** Super-Relaxed State (SRX), synthetic thick filaments (STFs), mavacamten, R403Q, interacting-heads motif (IHM), Bc, bovine cardiac, DMSO, dimethyl sulfoxide, DRX, disordered relaxed, ELC, essential light chain, HCM, hypertrophic cardiomyopathy, HMM, heavy meromyosin, IHM, interacting-heads motif, LMM, light meromyosin, MyBPC, myosin-binding protein C, RLC, regulatory light chain, S1, subfragment 1, S2, subfragment 2, SRX, super-relaxed, STFs, synthetic thick filaments

## Abstract

A hallmark feature of myosin-II is that it can spontaneously self-assemble into bipolar synthetic thick filaments (STFs) in low-ionic-strength buffers, thereby serving as a reconstituted in vitro model for muscle thick filaments. Although these STFs have been extensively used for structural characterization, their functional evaluation has been limited. In this report, we show that myosins in STFs mirror the more electrostatic and cooperative interactions that underlie the energy-sparing super-relaxed (SRX) state, which are not seen using shorter myosin subfragments, heavy meromyosin (HMM) and myosin subfragment 1 (S1). Using these STFs, we show several pathophysiological insults in hypertrophic cardiomyopathy, including the R403Q myosin mutation, phosphorylation of myosin light chains, and an increased ADP:ATP ratio, destabilize the SRX population. Furthermore, WT myosin containing STFs, but not S1, HMM, or STFs-containing R403Q myosin, recapitulated the ADP-induced destabilization of the SRX state. Studies involving a clinical-stage small-molecule inhibitor, mavacamten, showed that it is more effective in not only increasing myosin SRX population in STFs than in S1 or HMM but also in increasing myosin SRX population equally well in STFs made of healthy and disease-causing R403Q myosin. Importantly, we also found that pathophysiological perturbations such as elevated ADP concentration weakens mavacamten’s ability to increase the myosin SRX population, suggesting that mavacamten-bound myosin heads are not permanently protected in the SRX state but can be recruited into action. These findings collectively emphasize that STFs serve as a valuable tool to provide novel insights into the myosin SRX state in healthy, diseased, and therapeutic conditions.

Vertebrate striated muscle contraction results from cyclic interactions between myosin heads on the thick filaments and actin monomers on the thin filament. Emerging evidence in the last decade suggests that, in addition to Ca^2+^-mediated regulatory mechanisms within the thin filaments, the strength of muscle contraction may also be tuned by mechanisms intrinsic to myosin on the thick filaments (1, 2, 3, 4, 5, 6). Specifically, myosin heads in relaxed thick filaments are thought to exist in an equilibrium between two functional states (7, 8, 9): (1) the disordered relaxed (DRX) state, in which myosin is free and ready to interact with actin and has an average ATP turnover time of <10 s and (2) the super-relaxed (SRX) state, in which myosin is unavailable for interaction with actin and has a prolonged ATP turnover time of >100 s. Many studies have started showing that the availability of the myosin heads to form actin cross bridges can be controlled by altering this DRX–SRX equilibrium, which in turn can regulate the strength of striated muscle contraction, all of which has been discussed in a recent report by Nag and Trivedi (7).

The original discovery of the SRX state was based on single ATP turnover experiments in skinned rabbit fast and slow skeletal muscles (10), which was later confirmed by studies in skeletal and cardiac muscle systems from other species (8, 11, 12, 13, 14, 15, 16, 17, 18). The structural basis for this biochemical SRX state is unclear because of a lack of high-resolution atomic-level structure of the myosin. At best, the functional SRX state of myosin has been loosely correlated to the structural interacting-heads motif (IHM) state of myosin in which the two myosin heads assymetrically interact with one another while folding back onto the proximal tail (1, 2, 3). In the muscle thick filaments, it has been hypothesized that, in addition to myosin interactions with titin and myosin-binding protein C (MyBPC), the folded-back myosin IHM state is stabilized by several intramolecular interactions among various subdomains of myosin such as the regulatory light chain (RLC), heavy meromyosin (HMM), light meromyosin (LMM), subfragment 1 (S1), and subfragment 2 (S2) that give rise to S1–S1, S1–S2, RLC–RLC, S1–LMM, and S2–LMM interactions (1, 2, 3). If these interactions underlying the IHM state also give rise to the functional SRX state, then it is essential to build an experimental model that can capture most of these features. In previous studies, shorter myosin models such as full-length HMM, 25-hep HMM (HMM containing 25 heptad repeats of the S2), 2-hep HMM (HMM containing 2 heptad repeats of the S2), and S1 were all shown to form the SRX state (13, 19). However, in comparison to 2-hep HMM or S1, not only the SRX population was higher in full-length HMM and 25-hep HMM, but also the DRX–SRX equilibrium was more sensitive to electrostatic perturbations (13, 19). These observations suggested that the myosin SRX state is more stable in the presence of two S1 heads and the extended portion of the proximal tail. Importantly, myosin under physiological conditions does not exist in these short forms but present in an extensive filamentous form formed by the coiled-coil interactions of distal tails, highlighting an essential role for the extended tail in the myosin function. The present study focuses on using reconstituted thick filaments that mimic the structural environment close to the native thick filaments for functional characterization of myosin in an in vitro setting.

There are two different ways to study myosin thick filaments. The first, called the native thick filaments, involves thick filaments extracted from the native muscle by removing the thin filaments using gelsolin treatment (20, 21), and the second, referred to as the reconstituted or synthetic thick filaments (STFs), involves spontaneous self-assembly of myosin into bipolar thick filaments by lowering the ionic strength of the buffer containing full-length myosin (22, 23). Of these two, the first model provides a native-like environment to study the myosin thick filament in the presence of MyBPC, titin, and other thick filament–associated proteins. The latter, STFs, represents a simpler model that captures essential interactions pertinent to myosin alone while retaining the 14.3-nm myosin subunit periodicity and a 43-nm axial periodicity as in native filaments (24, 25). Also, electron microscopy structures confirm a bipolar myosin arrangement and the bare zone in both native thick filaments and STFs (22, 26, 27). In addition, the 3-fold rotational symmetry found in native thick filaments is also preserved in STFs (28). The STF model also provides a bottom-up approach to study the underlying biology of thick filaments and their function, starting from full-length myosin, offering advantages in different contexts. For example, this model permits the controlled addition of binding partners such as MyBPC to build a more complex system (29). Also, this system allows us to recombinantly study mutations in the rod domain of the myosin that causes several skeletal and cardiac myopathies (30, 31). Finally, different studies have proposed cooperative myosin activation in thick filaments by studying several physiological perturbations such as an increased ADP:ATP ratio (10, 15, 32), RLC phosphorylation (3), strong actin binding of myosin and Ca^2+^ binding to thick filaments (33, 34, 35), which are important physiological aspects that can be easily studied in a system containing assembled myosin filaments that preserves most of the myosin molecular interactions typically present in thick filaments. However, the use of such reconstituted thick filaments for functional studies, especially in relevance to the low energy–consuming SRX state of myosin, has been sparse.

In this study, using shorter cardiac myosin models such as HMM and S1 as well as STFs made of cardiac full-length myosin, we tested the hypothesis that several intramolecular interactions within the myosin molecule that are known to stabilize the IHM state of myosin may also underlie the SRX state. We provide evidence that many electrostatic and cooperative interactions that underlie the myosin SRX state are preserved in STFs than in HMM and S1. Using STFs, we then show that many pathophysiological insults of hypertrophic cardiomyopathy (HCM) such as R403Q myosin mutation, hyper-phosphorylation of myosin light chains, and increased ADP:ATP ratio destabilize the myosin SRX population. Interestingly, such destabilizing effect of ADP on the SRX state is only seen in STFs made of WT myosin, but not in S1, HMM, or STFs made of mutant R403Q myosin. In addition, we show that a cardiac myosin inhibitor, mavacamten (36, 37), which is currently in phase III clinical trials for treating HCM, reduces the cardiac muscle contractility by shifting the myosin DRX–SRX equilibrium more towards the SRX state, consistent with some previous reports (13, 19, 38). However, using concentration-dependent responses in this study, we show that this ability of mavacamten to promote myosin into the SRX state is greater in STFs than in HMM and S1. We also found that the potency of mavacamten in promoting myosin into the SRX state is similar in STFs made of healthy atrial WT, healthy ventricular WT, or disease-causing ventricular mutant (R403Q) myosins. We further demonstrate that the mavacamten-bound SRX heads in STFs can be reversibly recruited into the DRX state *via* the ADP-mediated cooperative activation of thick filaments. This mechanism is not reproduced in either HMM or S1.

## Results

### STFs show similar steady-state basal myosin ATPase activity compared to S1 and HMM

**Figure 1:**
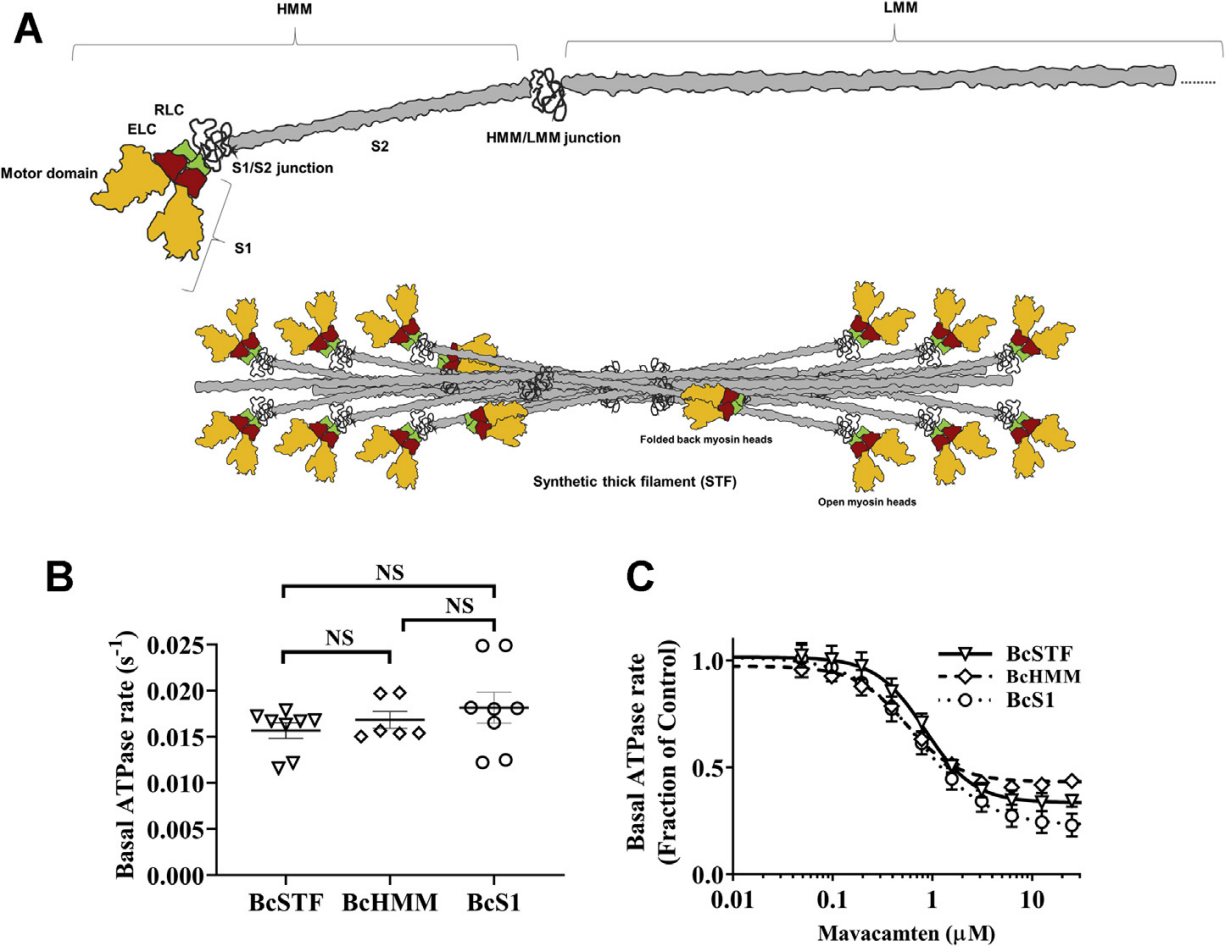
**Basal myosin ATPase activity, as well as potency of mavacamten inhibition in the basal myosin ATPase activity, is similar between BcSTFs, BcHMM, and BcS1.***A*, the schematic structure of the cardiac myosin with the different subdomains marked (*top*). The HMM domain of the myosin used in this study consists of the two S1 domains and the S2 domain. The S2 domain of an HMM molecule prepared by chymotryptic cleavage of myosin contains ∼46 heptad repeats. A previous study by Anderson *et al*. (13) used 2 and 25 heptad versions of the HMM. The unstructured S1–S2 junction separates the S1 and the S2 subfragments of the myosin molecule. Each myosin S1 head consists of a motor domain, ELC, and RLC subunits. LMM is the C-terminal portion of the coiled-coil tail to the *right* of the HMM–LMM junction. A truncated version of the LMM is shown for illustrative purposes. Full-length myosin self-assembles into bipolar synthetic thick filaments (STFs) in low-ionic-strength buffer (<150 mM), with some myosins in the open-head configuration and others in the folded-back IHM-like state, with the S1 heads folded back onto the S2 tail (*bottom*). *B*, absolute basal ATPase rate (in *s*^−1^) of BcSTFs, BcHMM, and BcS1 before treatment with mavacamten. *C*, normalized basal ATPase profiles of BcSTFs, BcHMM, and BcS1 after treatment with increasing concentrations of mavacamten. BcS1 refers to bovine cardiac myosin subfragment-1, BcHMM refers to bovine cardiac heavy meromyosin, and BcSTFs refers to bovine cardiac synthetic thick filaments. The DMSO values were used to normalize the data in the respective untreated systems. The concentrations of mavacamten required for half-maximal change (IC_50_) estimated from these ATPase curves were 0.76 ± 0.06, 0.57 ± 0.06, and 0.82 ± 0.06 μM for BcS1, BcHMM, and BcSTFs, respectively. Data are expressed as the mean±SEM (*n* ≥ 6 from at least two experiments). ELC, essential light chain; HMM, heavy meromyosin; IHM, interacting-heads motif; LMM, light meromyosin; NS, not significant; RLC, regulatory light chain; S1, subfragment 1; S2, subfragment 2.

A schematic showing the full-length myosin structure with the different subdomains is shown in the top panel of Figure 1*A*. The HMM domain of the myosin used in this study was prepared by chymotryptic cleavage of full-length myosin and consists of the two S1 domains connected to the ∼46-heptad S2 domain. Each myosin S1 head consists of a motor domain, essential light chain (ELC), and RLC subunits. The S1 used in this study is the short S1 that includes the motor domain and the ELC but not the RLC. Full-length myosin reconstitutes into bipolar myosin filaments in a buffer of lower ionic strength (<150 mM) referred hereafter as STFs (bottom panel of Fig. 1*A*).

To evaluate whether bovine cardiac (Bc) shorter myosin fragments, S1 and HMM, differ from STFs in their biochemical properties, we first measured the basal ATPase activity in these myosin systems. The resulting measurements did not show significant differences among the three myosin systems (Fig. 1*B*). The basal ATPase activities for BcS1, BcHMM, and BcSTFs were 0.016 ± 0.02 (*n* = 8), 0.017 ± 0.001 (*n* = 6), and 0.018 ± 0.001 (*n* = 8) s^-1^, respectively. Similarly, the basal ATPases measured in other myosin models such as porcine cardiac STFs made of WT and R403Q myosin were 0.026 ± 0.001 (*n* = 12) and 0.023 ± 0.001 (*n* = 12), respectively. Next, we measured basal ATPase activity in various myosin systems in response to increasing concentrations of mavacamten. A comparison of these responses did not show any obvious differences between systems in the potency of mavacamten on myosin ATPase inhibition (Fig. 1*C*). Statistical analysis using one-way ANOVA confirmed that the concentrations of mavacamten required to attain IC_50_ in the basal ATPase activity were not significantly different between the three myosin groups. The IC_50_ values in BcS1, BcHMM, and BcSTFs were 0.76 ± 0.06 (*n* = 8), 0.57 ± 0.06 (*n* = 6), and 0.82 ± 0.06 μM (*n* = 8), respectively, which are consistent with those reported for S1 and HMM in previous reports (19, 36, 37). These results suggest that BcSTFs behaves the same way as BcHMM or BcS1 in the enzymatically coupled ATPase experiments .

### Ionic strength perturbations decrease the myosin SRX population more in STFs than in HMM and S1

It is well established that the IHM state of myosin, which could also underlie the SRX state, involves a complex set of intermolecular and intramolecular interactions that are electrostatic in nature (3, 39, 40). Therefore, in the next series of experiments, we perturbed these electrostatic interactions in BcS1, BcHMM, and BcSTFs by progressively increasing the ionic strength (KCl) of the buffer and examined the effect on parameters derived using single ATP turnover kinetic experiments. The fluorescence decay profile obtained during the chase phase in the single ATP turnover kinetic experiments characteristically depicted two phases, a fast phase followed by a slow phase (Fig. S1 in Supporting information). Therefore, a biexponential function was fitted to estimate four different parameters—A_fast_, *k*_fast_, A_slow_, and *k*_slow_—where A represents the % amplitude and *k* represents the observed ATP turnover rate of each phase (10, 13, 19). A_fast_ and *k*_fast_ characterize myosin in the DRX state, whereas A_slow_ and *k*_slow_ characterize it in the SRX state.

**Figure 2:**
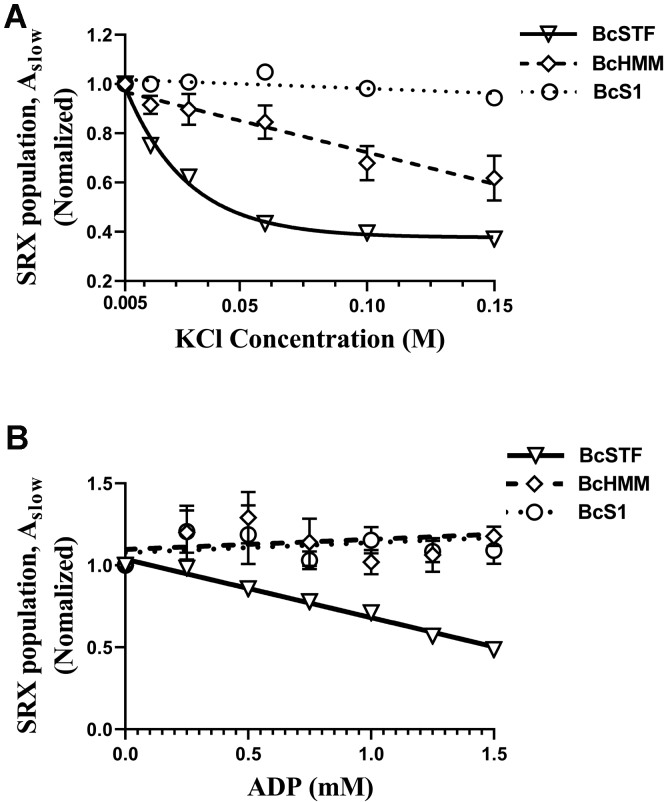
**Increasing KCl and ADP concentrations progressively decrease the myosin SRX population, particularly in BcSTFs.***A*, the effect of KCl on the myosin SRX population in various myosin models. *Symbols* represent the experimental data, and the corresponding best fits serve as a guide to the eye (linear for BcS1 and BcHMM but exponential for BcSTFs). Data are normalized to the myosin SRX values at 5-mM (0.005 M) KCl concentration in respective systems. *B*, the concentration-dependent effect of ADP on myosin SRX population in BcSTFs, BcHMM, and BcS1. In these experiments, care was taken to keep the total nucleotide (ATP + ADP) concentration to 2 mM in each case. Data are normalized to the myosin SRX values at the 0-mM ADP (2-mM ATP) in respective systems. The dependency of myosin SRX population on the ADP concentration was fitted to a linear regression model, and the resulting slopes were compared among groups. *Symbols* represent the experimental data, and the corresponding best fits (linear) serve as a guide to the eye. BcS1 refers to bovine cardiac myosin subfragment-1, BcHMM refers to bovine cardiac heavy meromyosin, and BcSTFs refers to bovine cardiac synthetic thick filaments. Data are expressed as the mean ± SEM (*n* ≥ 8 from at least two experiments). SRX, super-relaxed.

A qualitative comparison, as shown in Figure 2*A*, suggests that the response of BcSTFs and BcHMM to increasing KCl concentration was distinct from that of BcS1 (see Fig. S2, *A*-*C*, in Supporting information for representative raw traces). For example, A_slow_, which represents the % amplitude of the myosin SRX population, progressively decreased with increasing KCl concentration in both BcSTFs and BcHMM but not in BcS1. Furthermore, this dependency of myosin SRX population on KCl appeared to be steeper in BcSTFs than in BcHMM. To quantify the differences, we used best-fit relations as a guide to the eye. For example, the SRX–KCl relationships of BcS1 and BcHMM were better fitted to a straight line, whereas those of BcSTFs were better fitted to a monoexponential function. Following these relationships, KCl concentrations required to attain a 25% decrease in A_slow_ was 17 ± 1 mM in BcSTFs, 96 ± 22 mM in BcHMM, and >150 mM in BcS1. In contrast, myosin turnover rates in DRX (*k*_fast_) and SRX (*k*_slow_) states did not show any significant changes with ionic strength across all myosin types (Table S1 in Supporting information). Collectively, these findings suggest that the electrostatic interactions that underlie the myosin SRX state are more sensitive to electrostatic shielding in thick filaments than in shorter myosin fragments.

### Increasing the ADP:ATP ratio reduces the myosin SRX population in STFs but not in HMM and S1

Previous studies using single ATP turnover kinetics have shown that the slow phase (or the SRX phase) was diminished when Ca^2+^-activated cardiac and skeletal fibers incubated with mant-ATP were chased with nonfluorescent ADP (10, 15, 32). To examine whether this phenomenon can be reproduced in simpler myosin systems such as BcSTFs, BcHMM, and BcS1, we performed ADP chase experiments where we progressively increased ADP concentration from 0 to 2 mM in increments of 0.25 mM and assessed the relative changes in the myosin SRX population, A_slow_. Care was taken to ensure that the total nucleotide concentration (ATP + ADP) was 2 mM in each sample.

As shown in Figure 2*B*, the myosin SRX population, A_slow_, in BcSTFs gradually decreases with increasing ADP concentration, which is not observed in either BcHMM or BcS1 (see Fig. S2, *D*-*F*, in Supporting information for representative raw traces). To quantify the differences, we used slopes derived from a linear regression fit to individual myosin SRX–ADP relationships. Our analysis confirmed a negative linear correlation between A_slow_ and ADP concentration in BcSTFs with a slope of -0.35 ± 0.01 units that was statistically different from zero (*p* < 0.001). In contrast, BcHMM and BcS1 showed slopes of 0.12 ± 0.04 and 0.12 ± 0.05 units, both of which were not significantly different from zero (*p* = 0.34 for BcHMM and *p* = 0.44 for BcS1). One prominent structural feature of BcSTFs is the presence of a thick filament core, which is absent in both BcHMM and BcS1. Thus, a significant effect of ADP on destabilizing the myosin SRX population in BcSTFs appears to be strongly linked to this core (see Discussion). In contrast, myosin turnover rates in DRX (*k*_fast_) and SRX (*k*_slow_) states did not show any significant changes across all myosin types (Table S2 in Supporting information).

### Dephosphorylation of the myosin RLC and ELC increases the myosin SRX population

**Figure 3:**
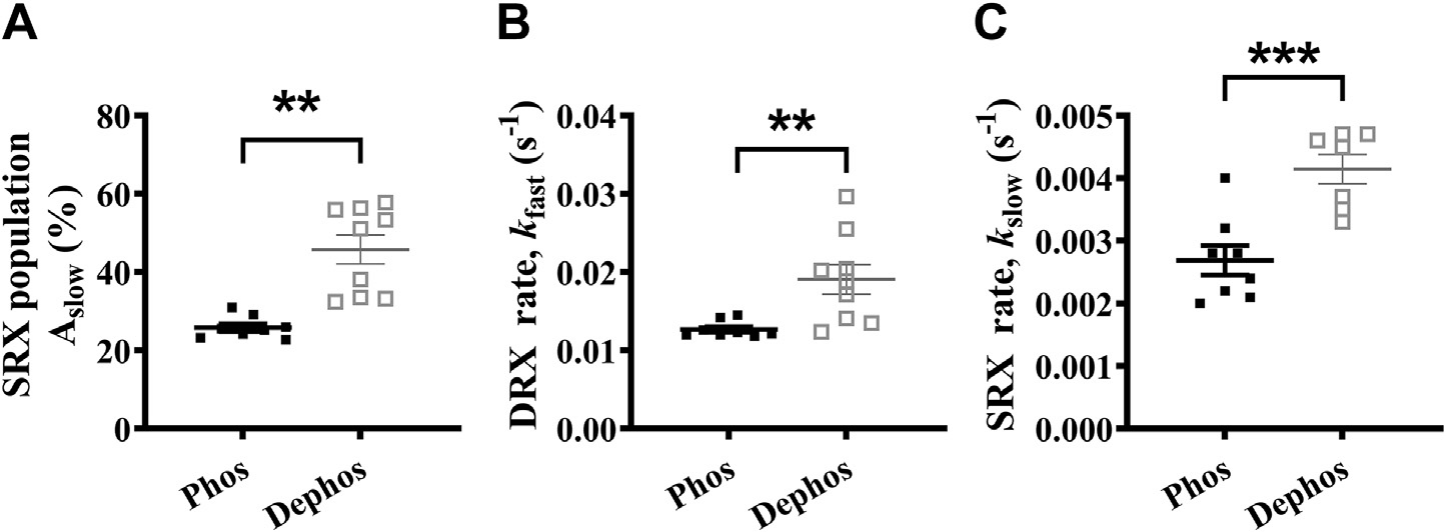
**Dephosphorylation of the myosin RLC and ELC increases the myosin SRX population.** Scatter plots showing the differences between untreated (with significant levels of phosphorylated ELC and RLC; Fig. S3*B* in Supporting information) and dephosphorylated myosin samples for (*A*) A_slow_, (*B*) *k*_fast_, and (*C*) *k*_slow_. Parameters are reported on an absolute scale (amplitudes in % and rates in s^-1^). Statistical differences were based on a two-tailed *t*-test (∗∗*p* < 0.01; ∗∗∗*p* < 0.001). Data are expressed as the mean ± SEM (*n* ≥ 4 for each). ELC, essential light chain; RLC, regulatory light chain; SRX, super-relaxed.

There is evidence to suggest that the changes in phosphorylation of the RLC can alter the DRX–SRX equilibrium (3). In contrast, the role of the ELC and the impact of its phosphorylation in this regard remain unclear. Here, we sought to examine the effect of a change in the RLC/ELC phosphorylation on the myosin SRX state. As described in Supporting information, we first ran a Pro-Q diamond gel to assess the phosphorylation status of RLC/ELC in a porcine cardiac full-length WT myosin sample, which confirmed that both RLC and ELC are phosphorylated to some extent in the basal state (Fig. S3*B* in Supporting information). We treated the myosin sample with lambda phosphatase, which significantly reduced the phosphorylation levels of both RLC and ELC, as evidenced by weak phospho-stained bands in the lambda phosphatase–treated sample (Fig. S3*B* in Supporting information). STFs made of lambda phosphatase–treated myosin showed a significant increase in A_slow_ by 20 ± 4% compared with STFs made of untreated myosin (Fig. 3*A*), suggesting that dephosphorylation of RLC/ELC shifts the myosin DRX–SRX equilibrium more towards the SRX state. This increase in A_slow_ is also associated with a 2-fold increase in both *k*_fast_ and *k*_slow_ (Fig. 3, *B*–*C*, and Table S3 in Supporting information), changes which may or may not be large enough to hold any biological significance.

### HCM-causing myosin R403Q mutation decreases the myosin SRX population

**Figure 4:**
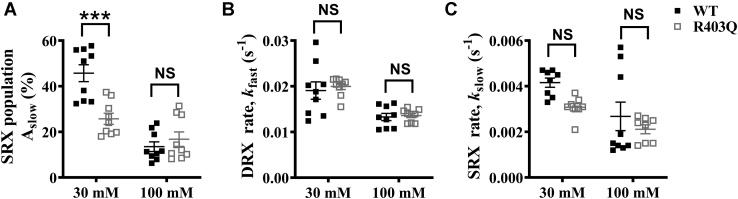
**HCM-causing R403Q myosin mutation destabilizes the myosin SRX state**. Scatter plots showing the effect of R403Q mutation at 30-mM and 100-mM KCl concentrations for (*A*) A_slow_, (*B*) *k*_fast_, and (*C*) *k*_slow_. PcSTF-WT and PcSTF-R403Q refer to porcine cardiac synthetic thick filaments made of WT and mutant R403Q myosin, respectively. All parameters are reported on an absolute scale (amplitudes in % and rates in s^-1^). Statistical differences presented are based on two-way ANOVA and subsequent post hoc Tukey’s pair-wise comparisons (∗∗∗*p* < 0.001). Data are expressed as the mean ± SEM (*n* ≥ 8 for each). HCM, hypertrophic cardiomyopathy; NS, not significant; SRX, super-relaxed.

There is now substantial evidence from purified recombinant myosin to muscle fiber studies that the HCM-causing R403Q mutation in myosin shifts the equilibrium of myosin heads from the SRX state to the DRX state (13, 38, 41). To investigate this possibility in the context of STFs, we generated STFs using full-length myosin isolated from porcine hearts containing a heterozygous R403Q mutation. The STFs made are, therefore, likely to contain an equal mixture of the WT and R403Q myosin, similar to what may be present in a clinical situation. Changes in RLC phosphorylation are known to occur in diseased hearts (42). Therefore, we first assessed the phosphorylation status of RLC and ELC in WT and R403Q myosin samples, as described in Supporting information. Our analysis showed that both the RLC and ELC are phosphorylated in either sample (see Fig. S3*B* in Supporting information). However, to avoid any contributions arising from slight differences in the phosphorylation status, we completely dephosphorylated the WT and R403Q myosin samples using lambda phosphatase to normalize the phosphorylation status of RLC/ELC before conducting experiments (see Fig. S3*B* in Supporting information). We then measured single ATP turnover kinetics in STFs made from lambda phosphatase-treated WT and R403Q myosin at two different KCl concentrations (30 and 100 mM) and assessed the differences in A_slow_, *k*_fast_, and *k*_slow_. Post hoc Tukey’s pair-wise comparisons from two-way ANOVA revealed that A_slow_ significantly decreased (*p* < 0.001) from 46 ± 4% in WT to 26 ± 3% in the R403Q group at 30-mM KCl, but it showed no significant change (*p* = 0.83) between groups at 100-mM KCl (Fig. 4*A*). In contrast, neither *k*_fast_ nor *k*_slow_ estimates were significantly different between groups at either KCl concentration (Fig. 4, *B*–*C*; Table S3 in Supporting information). Our data in PcSTFs verified that the R403Q mutation solely perturbs the SRX–DRX equilibrium and shifts this equilibrium more towards the DRX state, without affecting the intrinsic ATP turnover rate of myosin heads in the DRX and SRX states.

### ADP binding decreases the myosin SRX population in PcSTF-WT but not in PcSTF-R403Q

**Figure 5:**
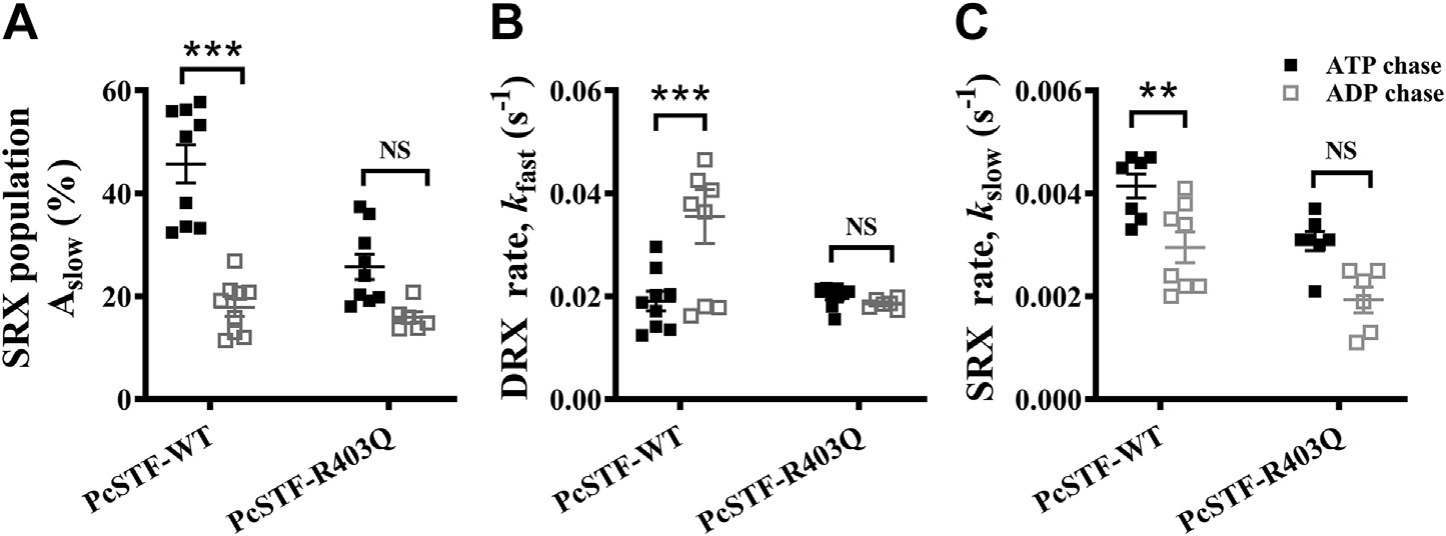
**ADP binding depopulates the myosin SRX state more in STF-WT than in STF-R403Q.** Scatter plots showing the effect of ATP chase versus ADP chase in PcSTF-WT and PcSTF-R403Q for (*A*) A_slow_, (*B*) *k*_fast_, and (*C*) *k*_slow_. All parameters are reported on an absolute scale (amplitudes in % and rates in s^-1^). PcSTF-WT and PcSTF-R403Q refer to porcine cardiac synthetic thick filaments made of WT and mutant R403Q myosin, respectively. Statistical differences presented are based on two-way ANOVA and subsequent post hoc Tukey’s pair-wise comparisons (∗∗*p* < 0.01; ∗∗∗*p* < 0.001). Data are expressed as the mean ± SEM (*n* ≥ 6 for each). NS, not significant; SRX, super-relaxed.

To examine whether the ADP-induced destabilization of the myosin SRX state is operative in an HCM disease model, we performed ADP chase experiments in PcSTF-WT and PcSTF-R403Q systems. Our analysis showed that A_slow_ in PcSTF-WT significantly decreased from 46 ± 4% when chased with ATP to 18 ± 1% when chased with ADP (*p* < 0.001; Fig. 5*A*), whereas this effect was not observed in PcSTF-R403Q (Fig. 5*A*). The decrease in A_slow_ observed in PcSTF-WT after ADP chase also coincided with a small but significant increase in *k*_fast_ and a significant decrease in *k*_slow_ (Fig. 5, *B–*C, and Table S3 in Supporting information). However, as argued above, these changes in rates are not large enough and may or may not hold any biological relevance. Interestingly, a similar comparison on the effect of ADP on A_slow_ among bovine systems (BcS1, BcHMM, and BcSTFs) containing WT myosin confirmed that the ADP destabilizes myosin SRX population in STFs, but not in BcS1 and BcHMM (Fig. S4 and Table S3 in Supporting information). Collectively, these observations indicate that the ADP-bound myosin heads also shift the DRX–SRX equilibrium more towards the DRX state. However, this effect requires a reserve in the SRX population and the myosin thick filament core, both of which are present in PcSTF-WT but not in BcHMM, BcS1, and PcSTF-R403Q (see Discussion).

### Mavacamten populates myosin in the SRX state more effectively in STFs than in HMM and S1

**Figure 6:**
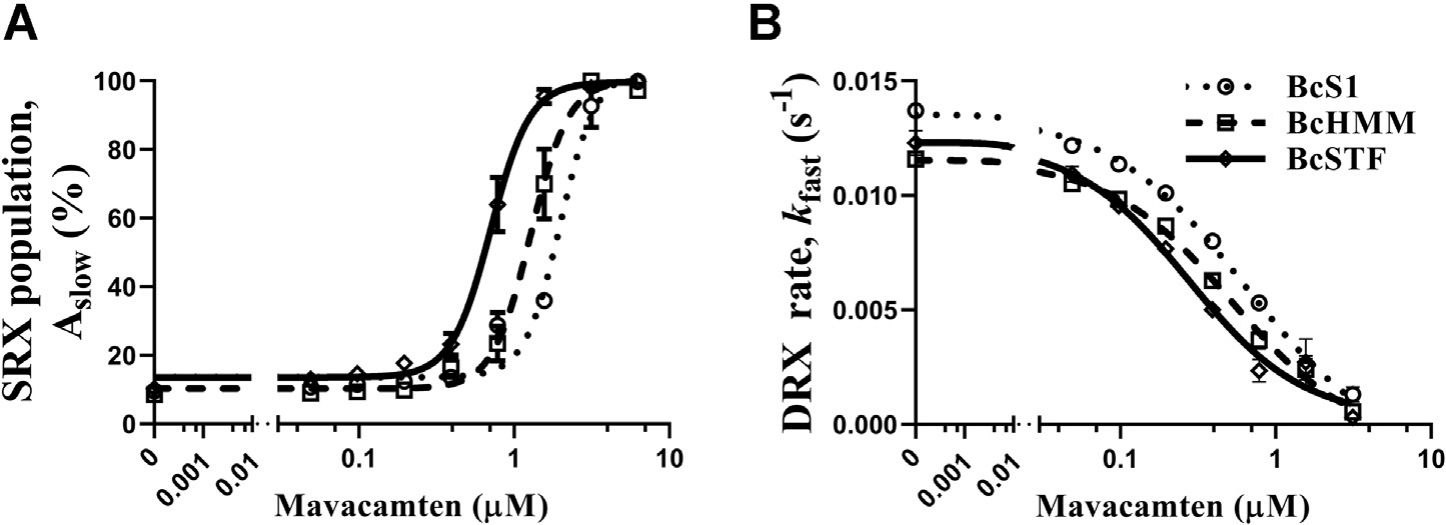
**Mavacamten populates myosin in the SRX state more effectively in STFs than in HMM and S1.** Comparison of the responses of mavacamten between BcS1, BcHMM, and BcSTFs for (*A*) A_slow_ and (*B*) *k*_fast_. BcS1 refers to bovine cardiac myosin subfragment-1, BcHMM refers to bovine cardiac heavy meromyosin, and BcSTFs refers to bovine cardiac synthetic thick filaments. Data are expressed as the mean ± SEM (*n* = 12 from three experiments). In panels *A* and *B*, concentrations of mavacamten required for a half-maximal increase in A_slow_ (EC_50_) for BcSTFs, BcHMM, and BcS1 were 0.66 ± 0.05, 1.17 ± 0.13, and 2.16 ± 0.19 μM, whereas those required for the half-maximal decrease (IC_50_) in *k*_fast_ were 0.31 ± 0.05, 0.54 ± 0.08, and 0.67 ± 0.09 μM, respectively. SRX, super-relaxed.

Previous studies have shown that mavacamten shifts the cardiac myosin DRX–SRX equilibrium towards the SRX state (13, 19, 38). Here, for the first time, we examined the concentration-dependent effect of mavacamten on the myosin SRX state in STFs relative to S1 and HMM. Compared to BcS1, A_slow_ profiles showed a more prominent leftward shift in BcSTFs than in BcHMM (Fig. 6*A* and Fig. S5 in Supporting information), suggesting a lower concentration of mavacamten is required to attain the same increase in myosin SRX population in BcSTFs than in BcHMM. For example, at a mavacamten concentration of 1.56 μM, the respective increases in A_slow_ in BcSTFs, BcHMM, and BcS1 were 98 ± 2%, 70 ± 10%, and 36 ± 1%, confirming a more prominent effect of mavacamten in STFs than in shorter myosin subfragments. This observation is also supported by the EC_50_ values derived for A_slow_, which were 0.66 ± 0.05, 1.17 ± 0.13, and 2.16 ± 0.19 μM for BcSTFs, BcHMM, and BcS1, respectively. Post hoc *t-*tests from one-way ANOVA confirmed that the EC_50_ of A_slow_ in BcSTFs is significantly lower by 2-fold (*p* < 0.05) when compared with BcHMM and by 3.3-fold (*p* < 0.001) when compared with BcS1. A similar trend was also noted in the ability of mavacamten in inhibiting *k*_fast_, the ATPase turnover rate of the myosin DRX heads (Fig. 6*B*). The IC_50_ values for *k*_fast_ in BcSTFs, BcHMM, and BcS1 were 0.31 ± 0.05, 0.54 ± 0.08, and 0.67 ± 0.09 μM, respectively (Table S4 in Supporting information). Collectively, these data indicate that the ability of mavacamten to promote more myosin into the SRX state is better realized in STFs than in shorter myosin models, S1 and HMM.

### The potency of mavacamten to populate myosin in the SRX state is lower in the presence of ADP

**Figure 7:**
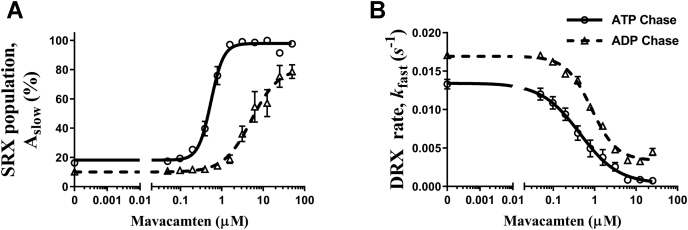
**The ability of mavacamten to populate myosin in the SRX state is weaker in the presence of ADP.** The effect of ATP versus ADP chase on (*A*) A_slow_ and (*B*) *k*_fast_ as a function of mavacamten. All experiments were performed using bovine cardiac synthetic thick filaments (BcSTFs). All parameters are reported on an absolute scale (amplitudes in % and rates in s^-1^). Data are expressed as the mean ± SEM (*n* ≥ 4 from two experiments). The concentrations of mavacamten required for half-maximal changes (EC_50_) in A_slow_ for ATP chase and ADP chase experiments were 0.63 ± 0.06 and >4.35 ± 0.64 μM, whereas those (IC_50_) for *k*_fast_ were 0.41 ± 0.06 and 1.14 ± 0.20 μM, respectively. SRX, super-relaxed.

We next examined the concentration-dependent effect of mavacamten on the SRX state in BcSTFs after ATP chase versus ADP chase to tie its mechanism to the biological function. Figure 7*A* shows that mavacamten populates myosin in the SRX state in a concentration-dependent manner, as evidenced by an increase in A_slow_. However, this ability to stabilize myosin heads in the SRX population was greatly diminished when chased with ADP (Fig. 7*A*). The concentrations of mavacamten required for the half-maximal increase in myosin SRX population in the ADP chase experiments were significantly higher by at least 7-fold (*p* < 0.001) than those in ATP chase experiments. The EC_50_ of A_slow_ in the ATP chase and ADP chase experiments were 0.63 ± 0.06 and >4.35 ± 0.64 μM, respectively (Table S4 in Supporting information). Similarly, the ability of mavacamten to inhibit the DRX ATPase rate was significantly (*p* < 0.05) reduced by 2.7-fold with ADP chase compared to ATP chase (Fig. 7*B*). The DRX ATPase IC_50_ values for ATP chase and ADP chase experiments were 0.41 ± 0.06 and 1.14 ± 0.20 μM, respectively (Table S4 in Supporting information). These results may hold significance in diseased conditions such as HCM, where a potential increase in the ADP concentration could add to the hypercontractile state of myosin (43).

### Mavacamten increases myosin SRX population equally well in healthy and diseased STFs

**Figure 8:**
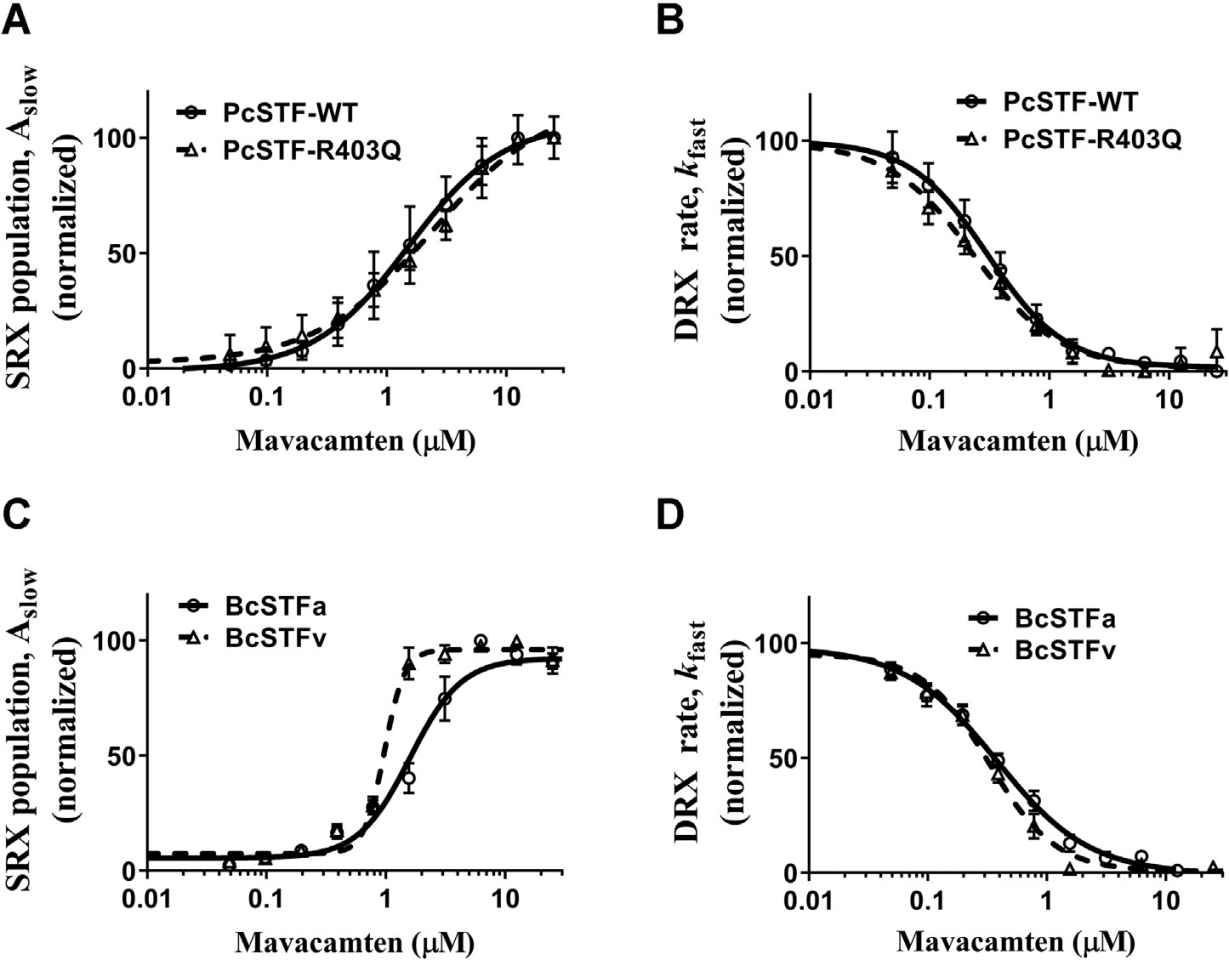
**Mavacamten populates myosin in the SRX state equally well in healthy and diseased models.** Comparison of the concentration-dependent effect of mavacamten between PcSTF-WT and PcSTF-R403Q for (*A*) A_slow_ and (*B*) *k*_fast_. Comparison of the effect of mavacamten between BcSTFs made from α-cardiac and β-cardiac myosin for (*C*) A_slow_ and (*D*) *k*_fast_. The nomenclature is as follows: PcSTFs and BcSTFs refer to porcine and bovine cardiac synthetic thick filaments, and BcSTFa and BcSTFv refer to bovine cardiac synthetic thick filaments made from left atrial (α-cardiac) and left ventricular (β-cardiac) full-length myosin, respectively. Data were expressed as the mean ± SEM (*n* ≥ 4 from two experiments). In panels *A* and *B*, the concentrations of mavacamten required for half-maximal change (EC_50_) in A_slow_ for WT and R403Q PcSTFs were 1.76 ± 0.22 and 2.00 ± 0.28, whereas those (IC_50_) for *k*_fast_ were 0.29 ± 0.02 and 0.30 ± 0.02 μM, respectively. Similarly, in panels *C* and *D*, the EC_50_ of A_slow_ for BcSTFa and BcSTFv were 1.83 ± 0.25 and 1.03 ±0.11 μM, whereas IC_50_ of *k*_fast_ were 0.61 ± 0.08 and 0.35 ± 0.06 μM, respectively. SRX, super-relaxed.

Next, we compared the effect of mavacamten in healthy (PcSTF-WT) and diseased (PcSTF-R403Q) in vitro model systems. As noted by changes in A_slow_, mavacamten increases myosin SRX population in a concentration-dependent manner with almost equal potency in either system (Fig. 8*A*). The concentrations required for the half-maximal increase in myosin SRX population were not statistically significant in WT and R403Q systems, and these were 1.76 ± 0.22 and 2.00 ± 0.28 μM, respectively. The increase in A_slow_ was associated with a concomitant decrease in *k*_fast_ with no significant differences between groups (Fig. 8*B*). The IC_50_ estimates of *k*_fast_ for WT and R403Q were 0.29 ± 0.02 and 0.30 ± 0.02 μM, respectively ( Table S4 in Supporting information), consistent with basal ATPase measurements (data not shown). These observations suggest that mavacamten can inhibit myosin activity by populating myosin in the SRX state equally well in a diseased model.

Recent studies have shown that in cellular models of MYH6 expressing the faster α-cardiac myosin, mavacamten inhibits myosin activity and populates the SRX state, similar to its effect in models expressing the slower β-cardiac myosin (38). However, there are no biochemical studies comparing the potency of mavacamten in these two systems side by side. To address this, we first purified full-length myosin from the left atrial and ventricular chambers of the bovine heart, and using a native PAGE gel, we confirmed that they predominantly express faster α-cardiac and slower β-cardiac myosin isoforms, respectively (Fig. S6 in Supporting information). We then made BcSTFs using these myosin variants (Fig. S3A in Supporting information) and conducted single turnover experiments in the presence of increasing concentrations of mavacamten. A comparison of concentration-dependent profiles of A_slow_ (Fig. 8*C*) and *k*_fast_ (Fig. 8*D*) suggests a slightly lower potency of mavacamten in increasing A_slow_ and in decreasing *k*_fast_ in BcSTFa (α-cardiac) than in BcSTFv (β-cardiac) systems. In support of this observation, our analysis confirmed that both the EC_50_ of A_slow_ and the IC_50_ of *k*_fast_ were significantly (*p* < 0.05) higher by 1.8-fold in BcSTFa than those in BcSTFv. The IC_50_ values of mavacamten for A_slow_ in BcSTFa and BcSTFv were 1.83 ± 0.25 and 1.03 ±0.11 μM, whereas those for *k*_fast_ were 0.61 ± 0.08 and 0.35 ± 0.06 μM, respectively (Fig. 8, *C–*D and Table S4 in Supporting information). These data are consistent with the observed inhibition in the basal ATPase assay among different myosin systems after mavacamten treatment (data not shown). Taken together, these data suggest that mavacamten is slightly more effective in enhancing the SRX state in β-cardiac than in α-cardiac myosin systems. Whether such small differences hold any relevance to clinical applications of mavacamten remains to be understood.

## Discussion

Muscle myosin-II is long known to spontaneously form bipolar STFs in buffers of low ionic strength (22, 23). With some caveats, such reconstituted thick filaments have been shown to structurally mimic essential features similar to the assembled myosin in native thick filaments (23). However, other than myosin assembly and disassembly kinetics, these STFs have found minimal use for functional studies of myosin (23, 44, 45, 46, 47, 48, 49). In this study, using STFs as our primary experimental model, we tested the hypothesis that the myosin folded-back IHM state, which is held by several intermolecular and intramolecular interactions between different subdomains of myosin, populates it in an energy-sparing myosin SRX state. Our findings demonstrate that STFs reproduce critical features, such as the cooperative behavior of the myosin SRX state, many of which are otherwise absent in the truncated myosin systems such as S1 and HMM.

Basal myosin ATPase assay demonstrated that the ATPase activity was similar among bovine cardiac STFs, HMM, and S1. The potency of mavacamten in inhibiting the basal myosin ATPase was also not different across these systems (Fig. 1, *B–C*). These results suggest that the overall ATPase activity of myosin was not altered in BcSTFs despite holding a more complex structure as compared with BcHMM and BcS1. However, it is worth noting that the ATP turnover rate measured in the ATPase assay represents the ensemble-averaged rate of myosin heads in the DRX and SRX states but does not quantify myosin population in these two states. Such quantification of myosin distribution in the SRX and DRX states requires single ATP turnover experiments.

It has been hypothesized previously that the biochemical SRX state with an ultra-low ATP turnover rate is primarily related to the IHM state (9, 11, 50, 51, 52), where the two myosin S1 heads interact with one another while asymmetrically folding back onto the proximal S2 tail, giving rise to several intramolecular interactions among various subdomains of myosin such as head–head (S1-S1), head–tail (S1-S2), RLC–RLC, S1–LMM, and S2–LMM (3). Given that all such possible interactions are present in STFs, a few of them in HMM, and none in S1, we sought to validate the role of these electrostatic interactions in stabilizing the SRX state of myosin. Not surprisingly, the myosin SRX population in STFs and HMM, but not in S1, progressively decreased in response to increasing ionic strength (Fig. 2*A*). More importantly, the relative magnitude of decrease in myosin SRX population is higher in STFs than in HMM for an equivalent increase in ionic strength (Fig. 2*A*). Our findings related to HMM and S1 are highly consistent with two previous studies (13, 19). Structurally, one crucial difference between myosin in STFs versus HMM is that the long distal tail of the full-length myosin in STFs can self-associate to form the thick filament backbone at low ionic strengths through intermolecular interactions such as S1-LMM, S2-LMM, LMM-LMM, and so forth. Therefore, our observations suggest that the distal tail of myosin that forms a thick filament backbone is essential to preserve all intermolecular and intramolecular interactions that stabilize myosin in the SRX population. This was also evident in the report by Anderson *et al*. (13), where the myosin SRX population in muscle fibers was higher than in purified proteins despite the higher ionic strength used in these experiments. Similarly, consistent with other studies (10, 12, 34, 53), we show that RLC phosphorylation destabilizes the myosin SRX population in STFs (Fig. 3), further suggesting that electrostatic interactions play a crucial role in holding the myosin population in the SRX and IHM states. It is worth noting in this context that, although S1 is insensitive to increasing ionic strength, perhaps due to a lack of electrostatic interactions, our observations suggest that a small fraction (∼15–20%) of the myosin population in S1 does exist in the SRX state, consistent with two previously published works (13, 19). It has been argued previously (13) that a subpopulation of S1 molecules might assume a conformation in which the lever arm is tilted more towards the prestroke direction similar to that in the IHM structure, which could give rise to a low energy–consuming SRX state.

**Figure 9:**
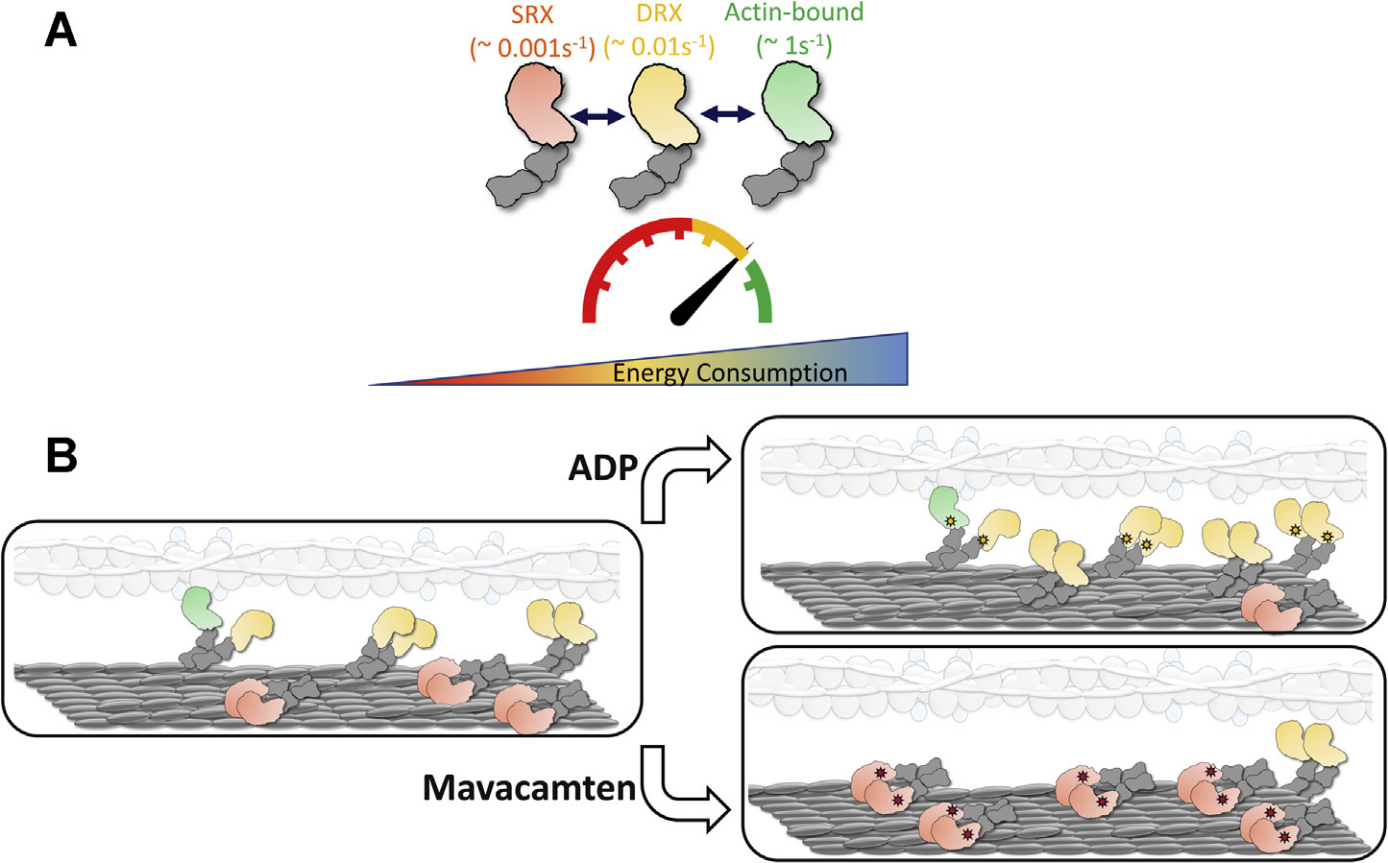
**Schematic representation of the different functional states of myosin and their possible distribution in the thick filament**. *A*, highlighted in *green* are actin-bound myosins, which are the most active (ATP turnover rate ∼ 1 s^−1^) and use maximum ATP. This myosin state is in equilibrium with an off-actin state (in *yellow*), the DRX state of myosin, which has 100-fold less activity (0.01s^−1^) than the actin-interacting form. Myosins in the DRX state are in equilibrium with those in the SRX state (in *red*), which has 10-fold lower activity (0.001s^−1^) than the DRX state. *B*, shown on the *left* is the schematic representation of the distribution of these different myosin heads in the sarcomere. Shown on the *right* are two different perturbations: ADP (*top*) and mavacamten (*bottom*) that change the equilibrium of these myosin states. Binding of ADP (*yellow stars*) to myosin co-operatively destabilizes the myosin SRX state, possibly *via* a communication involving the myosin’s proximal and distal tails that form the thick filament backbone (*elongated gray oblong*). On the other hand, mavacamten binding to myosin (*red stars*) populates it more in the SRX state. The translucent image shown represents the thin filament system made of actin filaments (*gray beaded circles*), tropomyosin (*elongated white oblong*) and troponin complex (*light blue circles*). DRX, disordered relaxed; SRX, super-relaxed.

Single-turnover experiments performed with slow skeletal, fast skeletal, cardiac, and tarantula muscle fibers have shown disruption of the myosin SRX state in the presence of ADP (10, 15, 32). At least with the skeletal muscle fibers, this observation has also been qualitatively recapitulated when studied at very long sarcomere lengths where there is no overlap between actin and myosin, suggesting that the binding of ADP to some myosin heads cooperatively disrupts the SRX property of neighboring myosin heads through a pathway that resides within thick filaments. Very interestingly, such cooperative disordering (or activation) was suggested to be less prominent in cardiac than in fast skeletal muscle because ADP-bound myosin heads can fully depopulate the myosin population from the SRX to the DRX state in fast skeletal but not in cardiac muscle (10, 11). Consistent with this notion, STFs made of cardiac full-length myosin showed a progressive decrease in the myosin SRX population with an increasing ADP:ATP ratio but did not fully depopulate this state even at the highest ADP:ATP ratio (Fig. 2*B*, Fig. 5 and Fig. S4). More interestingly, this destabilization was only observed in STFs, but not in HMM or S1 (Fig. 2*B* and Fig. S4 in Supporting information), reinforcing the notion that the effect of ADP-bound myosin heads may likely involve cooperative activation of thick filaments. Such cooperative activation can only be achieved *via* a relay system that involves a communication between the proximal tails of myosin to the distal tails that form the thick filament backbone (Fig. 9), a pathway that is not present in HMM or S1. It is also worth noting that the single-turnover experiments presumably measure the phosphate release rate from myosin as this is the slowest step in the basal myosin ATPase cycle. In the presence of ADP, what may likely happen is that a fraction of myosins that have released the bound mant-nucleotides can now bind ADP, and the resulting structural changes in these myosins may, in turn, affect the neighboring myosins in the SRX state *via* cooperative mechanisms involving the thick filament core. Without this explanation, it is unlikely to expect a different outcome between ATP chase and ADP chase experiments in STFs.

In addition to demonstrating the cooperative nature of molecular interactions linked to the myosin SRX state in STFs, we show that STFs made of full-length myosin purified from the left ventricular muscle of heterozygous R403Q HCM pigs have less myosin in the SRX state relative to STFs made of WT myosin (Fig. 4). This is consistent with the behavior observed in purified myosin and muscle fibers containing the myosin R403Q mutation (13, 41). It has been suggested in the literature that the R403Q mutation in myosin lies in the putative interface between the two myosin heads. Hence, it may likely disrupt the SRX state by weakening the S1–S1 interaction but not by altering the S1–S2 interaction (39). Our results indicate that even at low ionic strength, R403Q weakens such S1–S1 interactions to facilitate myosin recruitment from the SRX to the DRX state, leaving behind a lower myosin SRX reserve that can be recruited by any mechanism. Consequently, STFs made of R403Q myosin did not elicit any significant decrease in the SRX population, whether it be due to an increase in ionic strength (Fig. 4*A*) or whether it be due to ADP chase (Fig. 5*A*). On the contrary, the initial SRX reserve in STFs made of WT myosin was higher. Therefore, the resulting decrease in the myosin SRX population in response to these perturbations was significantly greater (Fig. 4*A* and Fig. 5*A*).

It has been hypothesized that myosin heads in the SRX state act as a reserve and may be pulled into action in response to increased physiological demand (7, 8, 9). Along this line, it has been shown that several HCM-causing mutations in either myosin or MyBPC disrupt the SRX state and pull more myosin into play, resulting in clinical hypercontractility (7, 13, 17, 41, 54, 55, 56, 57). To negate this effect, MyoKardia, Inc. developed a small-molecule cardiac myosin inhibitor, mavacamten (36, 37), which at a single high concentration, has been previously shown to shift the myosin DRX–SRX equilibrium towards the SRX state, thereby increasing the myosin SRX reserve (13, 19, 38, 54). By studying the concentration-dependent effect of mavacamten, we show that it brings about inhibition in the basal ATPase activity (Fig. 1*B*) by primarily increasing the myosin SRX population in all myosin systems evaluated in this study including BcS1, BcHMM, and BcSTFs (Fig. 6). Our finding that mavacamten promotes a greater fraction of myosin into the SRX state in HMM than in S1 is consistent with recent reports (13, 19). Interestingly, this ability of mavacamten in promoting myosin into the SRX state (Fig. 9) is further enhanced in BcSTFs, reiterating the notion that all the key structural interactions that underlie the myosin SRX state are better preserved in STFs than in HMM or S1. These observations suggest that mavacamten is more effective in stabilizing the myosin SRX state in STFs than in HMM or S1. Our result that mavacamten can populate all S1 myosins into the SRX state is different from the conclusion of previous studies (13, 19), but is consistent with our observation that mavacamten completely inhibits the basal myosin ATPase activity in S1 (Fig. 1*B*). We note that in one such study (19), consistent with our observations, it was shown that the observed ATP turnover rate of the fast (DRX) phase decreases to 0.0038 s^-1^ in S1 treated with 30-μM mavacamten, which is close to the observed rate of the slow (SRX) phase (0.0075 s^-1^) in the untreated S1, bolstering the hypothesis that mavacamten can drive all the myosin heads in S1 into the SRX state.

To further demonstrate that mavacamten does not lock myosin heads permanently in the SRX state and that these heads can be recruited into the DRX state when required, we also performed ADP chase experiments (Fig. 7). Consistent with the activation mechanism detailed above for the ADP-bound myosin heads, mavacamten was less effective in increasing the myosin SRX population when chased with ADP at all concentrations (Fig. 7). This is substantiated by the 7-fold increase in the EC_50_ of mavacamten for myosin SRX population and 2.7-fold increase in the IC_50_ for DRX ATPase rate in ADP chase experiments relative to those in ATP chase experiments. An alternate explanation could be that mavacamten may bind weakly to myosin when the nucleotide pocket is occupied by ADP, explaining a lower SRX population independent of mavacamten concentration. Collectively, these observations made in STFs not only validate the inhibitory mechanism of mavacamten but also confirm that the mavacamten-bound heads can be reversibly recruited into the DRX state as needed. Such ADP-triggered activation of the muscle and the reduced SRX stabilization by mavacamten may be relevant in diseased conditions such as HCM or effects of inotropes that could potentially increase the ADP:ATP ratio (43).

For therapies involving the use of small-molecule drugs such as mavacamten, it is of considerable interest to evaluate the efficacy of these agents in a diseased setting. Earlier, we have shown that the HCM-causing R403Q mutation induces a hypercontractile function by shifting the myosin DRX–SRX equilibrium more towards the DRX state, presenting an excellent HCM disease model in which mavacamten can be tested as a proof of concept. When tested in this diseased model, we found that mavacamten is equally effective in populating the myosin SRX state in STFs made of WT and R403Q myosin (Fig. 8), consistent with a previous study showing the same but at a single high concentration of mavacamten (13). Similarly, we also found that mavacamten equally stabilizes the myosin SRX population in STFs made of WT atrial (α-cardiac) myosin and WT ventricular (β-cardiac) myosin. The physiological relevance of this finding in an in vivo setting remains to be clarified, and this has to await the results of future studies.

In summary, from observations made in this report, we infer here that reconstituted bipolar thick filaments formed from full-length myosin capture essential intermolecular and intramolecular interactions between various subdomains of myosin, which are essential to stabilize the myosin SRX population. It is reasonable to assume that the interplay of these complex molecular interactions is necessary while understanding the effects of physiological and pathological perturbations in the cardiac sarcomere. The use of such reconstituted bipolar thick filaments may be more valuable for studying the cooperative mechanisms within thick filaments, a topic that has been largely unexplored but gaining momentum in the last decade or so. For example, many biologically relevant perturbations such as Ca^2+^, ADP, disease-causing thick filament protein mutations, and possibly small molecules can modulate the cooperative mechanisms within thick filaments (Fig. 9), which can be better investigated using an experimental model that is close to the physiological setting. Therefore, the reconstituted bipolar thick filament model put forth here, although may not recapitulate the strain effect because of titin, it may serve as a valuable tool not only to better understand myosin structure–function relationships in a variety of contexts but may also aid us in the discovery of better therapeutic agents for disease prevention.

## Experimental procedures

### Cardiac protein preparations

Bovine cardiac actin was purified following a previously established method (58) with some modifications. Actin was stored at -80˚C as G-actin and polymerized fresh for each day of experiments by adding 50-mM KCl and 2-mM MgCl_2_ to the actin-containing buffer. β-cardiac full-length myosin from bovine and porcine left ventricles and α-cardiac full-length myosin from bovine left atrium were isolated following established methods described elsewhere (59). After this, proteins were dialyzed in a buffer containing 10-mM Pipes (pH 6.8), 300-mM KCl, 0.5-mM MgCl_2_, 0.5-mM EGTA, 1-mM NaHCO_3_, and 1-mM DTT and stored at −80 °C. Using bovine cardiac full-length myosin as the starting material, HMM and subfragment S1 were prepared according to methods described in a previous report (59).

### Reconstitution of myosin thick filaments

Full-length myosin remains fully soluble in a buffer of high ionic strength (300 mM). However, many previous studies had previously shown that the full-length myosin could spontaneously self-assemble into bipolar thick filaments when the ionic strength of the buffer was reduced to 150 mM or below (22, 23). This method was used to construct thick filaments with some modifications in this study. Briefly, the ionic strength of the myosin sample and the myosin concentration, were first adjusted to the desired value by diluting it with a buffer containing 20-mM Tris-HCl (pH 7.4), 0-mM KCl, 1-mM EGTA, 3-mM MgCl_2_, and 1-mM DTT. After dilution, the myosin sample was incubated for 2 h on ice to form thick filaments before using it for experiments. In this study, such reconstituted myosin filaments will be referred to as STFs. Alternatively, STFs were also made by slowly dialyzing full-length myosin into a low ionic (150 mM or below) strength buffer. Quantitatively, the measurements reported in STFs were not different between these two methods. Four different STF models were used in this study, including STFs made of porcine WT ventricular myosin (PcSTFs or PcSTF-WT), STFs made of porcine R403Q mutant ventricular myosin (PcSTF-R403Q), STFs made of bovine WT ventricular myosin (BcSTFs or BcSTFv), and STFs made of bovine WT atrial myosin (BcSTFa).

### Steady-state ATPase measurements

Measurements of basal myosin ATPase activity in BcSTFs, BcHMM, and BcS1 (chymotryptic S1 without the RLC) systems as a function of increasing mavacamten concentrations were performed at 23 °C on a plate-based reader (SpectraMax 96-well) using an enzymatically coupled assay (37). The composition of the buffer used for these experiments was 12-mM Pipes (pH 6.8), 2-mM MgCl_2_, 10-mM KCl, and 1-mM DTT. A concentrated stock (20 mM) of mavacamten, synthesized by MyoKardia, Inc., was first prepared using dimethyl sulfoxide (DMSO), and this was used to attain the desired concentration ranging from 0 to 50 μM in the final buffer samples. A final DMSO concentration of 2% was achieved in all samples. Data recorded by the instrument-compatible software package, SoftMax Pro, was exported to GraphPad Prism and analyzed. A final myosin concentration of 1 μM was attained irrespective of the myosin model used.

### Single mant-ATP turnover kinetics

Single ATP turnover kinetic experiments using a fluorescent 2′/3′-O-(N-Methylanthraniloyl) (mant)-ATP were conducted on two different instruments, a stopped-flow unit, and a 96-well plate fluorescence plate reader. A protocol involving the same two-step mixing, as detailed in previous studies (10, 13, 19), was executed on both instruments. This assay measures fluorescent nucleotide release rates after incubation of myosin preparations with mant-ATP and chased with excess unlabeled ATP. The myosin kinetics of this fluorescent mant-ATP is known to be very similar to that of the nonfluorescent ATP (60). For the plate-based method, in the first step, 100 μl of 0.8-μM myosin was combined with 50 μl of 3.2-μM mant-ATP in a UV-transparent fluorescence plate and the reaction was aged for 60 s to allow binding and hydrolysis of mant-ATP to inorganic phosphate and mant-ADP. In the second step, mant-nucleotides were chased with nonfluorescent ATP by adding 50 μl of 16-mM nonfluorescent ATP to the above mixture, and the resulting fluorescence decay due to mant-nucleotide dissociation from myosin was monitored over time. The composition of the final buffer was as follows: 20-mM Tris-HCl (pH 7.4), 30-mM KCl, 1-mM EGTA, 3-mM MgCl_2_, and 1-mM DTT. The concentrations of myosin, mant-ATP, and nonfluorescent ATP attained in the final mixture were 0.4 μM, 0.8 μM, and 4 mM, respectively. The final concentration of DMSO was 2% in all samples. The excitation wavelength for monitoring the mant-nucleotides was 385 nm. The emission was acquired using a long-pass filter with a cutoff at 450 nm. Data were acquired at a frequency of 4.25 Hz for the first 500 points, followed by 1 Hz for the next 430 points and finally at 0.16 Hz for the rest of the duration. All experiments were performed at 25 °C. In addition to ATP chase experiments, we also acquired fluorescence decay profiles using the ADP chase. This experiment was carried out in the same way as the ATP chase experiment except that nonfluorescent ADP was used in the place of nonfluorescent ATP to chase mant-nucleotides in the second step.

Consistent with similar studies in the past (10, 13, 19), the fluorescence decay profile obtained during the chase phase characteristically depicts two phases, a fast phase followed by a slow phase (Fig. S1 in Supporting information). Therefore, a biexponential function was fitted to each trace to estimate four parameters corresponding to fast and slow phases—A_fast_, *k*_fast_, A_slow_, and *k*_slow_—where A represents the normalized % amplitude and *k* represents the observed ATP turnover rate of each phase (Fig. S1 in Supporting information). In this study, the fast and the slow phases correspond to the myosin activity in the DRX and SRX states, respectively.

### Data analysis

For each assay, the experiment was repeated at least twice with a minimum of two replicates per experiment. Sample number *n* refers to the total number of measurements made (the number of experiments ∗ the number of replicates), which were averaged and presented as the mean ± SEM. Dose-response profiles obtained using mavacamten were used to estimate the concentration (in μM) required to attain half-maximal change (EC_50_ or IC_50_) in a given parameter. In experiments involving only one factor, we used a two-tailed student’s *t*-test. For experiments involving two factors, we used a two-way ANOVA to analyze the effect of both factors. Subsequent post hoc *t*-tests using Tukey’s pair-wise comparisons were used to statistically differentiate the changes in parameters among groups. Significance was set at *p* < 0.05.

## Data availability

All data presented in this manuscript are either included in the main article or the Supporting Information.

## Conflict of interest

S. K. G., M. Y., Q.-F. G., and S. N. are all employees of MyoKardia, Inc. and hold company shares through their employment. The authors declare that they have no conflicts of interest with the contents of this article.
