# Supplementary material for: Synthetic thick filaments: A new avenue for better understanding the myosin super-relaxed state in healthy, diseased, and mavacamten-treated cardiac systems
Source: J Biol Chem. 2020 Dec 3;296:100114. doi: 10.1074/jbc.RA120.016506 (PMC7948491; doi:10.1074/jbc.RA120.016506)
Supplement: Figures and Tables [file mmc1.pdf]

SUPPORTING INFORMATION

Synthetic thick filaments: A new avenue for better understanding the myosin super-relaxed state in healthy, disease, and mavacamten-treated cardiac systems

Sampath K. Gollapudi, Ming Yu, Qing-Fen Gan, and Suman Nag[†]
Department of Biology, MyoKardia, Inc., Brisbane, CA 94005

Corresponding author: Suman Nag
E-mail: snag@myokardia.com

Running title: Super-relaxed state in reconstituted myosin thick filaments

Keywords: Super-Relaxed State (SRX), Synthetic thick filaments (STFs), mavacamten, R403Q, Interacting-Heads Motif (IHM)

MATERIALS AND METHODS

Determination of Phosphorylation Status of Thick Filament Proteins

The concentrations of porcine wild-type (WT) and mutant (R403Q) full-length myosin samples were first adjusted to 2 mg/ml in a buffer containing Pipes (pH 6.8), 300 mM KCl, 0.5 mM $MgCl_2$, 0.5 mM EGTA, 1 mM $NaHCO_3$, and 1 mM DTT. Each sample was treated with an appropriate amount of lambda phosphatase supplemented by $MnCl_2$ and Protein MetalloPhosphatases, as detailed in the manufacturer's protocol (Catalog #P0753L, New England Biolabs, Ipswich, MA). Following treatment, samples were kept at room temperature for 30 minutes and then left at 4°C overnight to allow complete dephosphorylation. For analyzing the phosphorylation status of proteins, equal volumes (10μl) of various samples were loaded and run on a Mini-PROTEAN Tris-Tricine precast SDS-gels (Bio-Rad, Hercules, CA), following which the gel was treated with Pro-Q diamond stain and destained as described in the manufacturer's manual (P33300 and P33310; Life Technologies, Carlsbad, CA). The phosphorylation status of proteins on the gel was visualized by irradiating it with fluorescent light. The gel was stained using Coomassie brilliant blue (R-250; Bio-Rad Laboratories, Hercules, CA) to visualize the total protein amounts. Densitometric analysis of the resulting protein profiles was performed using the ImageJ software (available on the National Institutes of Health website; https://imagej.nih.gov/ij/) to compare the phosphorylation status of proteins among WT and R403Q myosin before and after treatment with lambda phosphatase.

SUPPLEMENTAL RESULTS

SDS-PAGE and Phosphorylation Analysis of Myosin Proteins

An overloaded Coomassie blue-stained SDS-gel of different myosin systems including bovine ventricular full-length myosin, bovine atrial full-length myosin, bovine cardiac heavy meromyosin (HMM), and bovine subfragment-1 (S1) is shown in Fig. S3A. The purity of full-length myosin produced using methods discussed in this study is usually > 95%. The digested smaller versions of myosins (HMM and S1) had a few extra bands, possibly due to loop cuts in the myosin structure.

The proteins that undergo phosphorylation in myosin are the regulatory light chain (RLC) and the essential light chain (ELC). A representative gel shown in Fig. S3B confirms noticeable phosphorylation of the RLC and the ELC in both wild-type and R403Q samples before treatment with lambda phosphatase. To ensure that the results presented in this study are not affected by small differences in the phosphorylation of RLC/ELC between groups, we dephosphorylated samples using lambda phosphatase. Fig. S3B confirms near-complete dephosphorylation in both WT and R403Q myosin.

Table S1. Parameters derived using single ATP turnover experiments in various myosin groups in response to increasing KCl concentration

**Parameter**	**[KCl]**	**Ionic Strength**	**BcSTFs**	**BcHMM**	**BcS1**
SRX population, A_{slow} (%)	5 mM	32.5 mM	26.76±0.37	31.07±2.85	19.97±0.54
	15 mM	42.5 mM	20.03±0.48	29.97±2.04	19.97±0.78
	30 mM	57.5 mM	16.59±0.40	27.18±1.60	20.12±0.63
	60 mM	87.5 mM	11.10±0.31	26.21±1.41	20.94±0.67
	100 mM	127.5 mM	10.55±0.46	20.76±2.22	19.62±0.51
	150 mM	177.5 mM	9.90±0.66	18.35±2.11	18.82±0.33
DRX ATPase rate, k_{fast} (s^{-1})	5 mM	32.5 mM	0.0108±0.0003	0.0058±0.0004	0.011±0.002
	15 mM	42.5 mM	0.0098±0.0001	0.0090±0.0009	0.011±0.001
	30 mM	57.5 mM	0.0093±0.0002	0.0093±0.0008	0.011±0.001
	60 mM	87.5 mM	0.0082±0.0002	0.0079±0.0005	0.009±0.001
	100 mM	127.5 mM	0.0081±0.0002	0.0092±0.0006	0.008±0.001
	150 mM	177.5 mM	0.0071±0.0001	0.0087±0.0004	0.007±0.001
SRX ATPase rate, k_{slow} (s^{-1})	5 mM	32.5 mM	0.0018±0.0001	0.0008±0.0004	0.0008±0.00005
	15 mM	42.5 mM	0.0015±0.0001	0.0009±0.0005	0.0009±0.00006
	30 mM	57.5 mM	0.0012±0.0001	0.0006±0.0003	0.0009±0.00002
	60 mM	87.5 mM	0.0008±0.0001	0.0005±0.0003	0.0008±0.00003
	100 mM	127.5 mM	0.0007±0.0001	0.0010±0.0004	0.0008±0.00002
	150 mM	177.5 mM	0.0004±0.0001	0.0007±0.0004	0.0007±0.00003

Nomenclature is as follows: BcSTFs, bovine cardiac synthetic thick filaments; BcS1, bovine cardiac myosin sub-fragment S1; BcHMM, bovine cardiac heavy meromyosin; The ionic strength at different buffer conditions has been calculated as $I = \frac{1}{2}\sum_i C_i z_i^2$, assuming complete ionization of the Tris-HCl, $MgCl_2$, and KCl components in the buffer system. C_i is the concentration of the ith ionized species with a charge of z_i. Data are reported as mean±SEM (n≥4 from two experiments for each group). All myosin samples used here were not subjected to dephosphorylation.

Table S2. Parameters derived using single ATP turnover experiments in various myosin groups in response to increasing ADP concentration

Parameter	[KCl]	[ADP]	[ATP]	BcSTFs	BcHMM	BcS1
SRX population, A_{slow} (%)	30 mM	0.00 mM	2.00 mM	21.0±0.8	9.7±1.1	8.3±0.5
		0.25 mM	1.75 mM	20.6±0.9	11.5±2.0	10.3±1.7
		0.50 mM	1.50 mM	17.8±0.9	9.9±0.9	10.3±2.2
		0.75 mM	1.25 mM	16.5±0.4	10.3±1.3	8.5±0.6
		1.00 mM	1.00 mM	14.9±0.6	8.9±0.6	9.2±0.4
		1.25 mM	0.75 mM	12.0±0.5	9.7±0.9	8.9±0.4
		1.50 mM	0.50 mM	10.4±0.6	12.5±1.4	8.9±0.7
		1.75 mM	0.25 mM	8.9±0.3	14.4±1.9	12.2±1.3
DRX ATPase rate, k_{fast} (s^{-1})	30 mM	0.00 mM	2.00 mM	0.019±0.0027	0.012±0.0004	0.015±0.0003
		0.25 mM	1.75 mM	0.015±0.0002	0.013±0.0004	0.015±0.0003
		0.50 mM	1.50 mM	0.017±0.0002	0.012±0.0005	0.014±0.0003
		0.75 mM	1.25 mM	0.019±0.0004	0.013±0.0004	0.014±0.0004
		1.00 mM	1.00 mM	0.021±0.0004	0.012±0.0005	0.014±0.0004
		1.25 mM	0.75 mM	0.025±0.0013	0.012±0.0004	0.014±0.0003
		1.50 mM	0.50 mM	0.027±0.0007	0.013±0.0005	0.014±0.0005
		1.75 mM	0.25 mM	0.027±0.0020	0.014±0.0013	0.015±0.0005
SRX ATPase rate, k_{slow} (s^{-1})	30 mM	0.00 mM	2.00 mM	0.0027±0.0003	0.0020±0.0001	0.0016±0.0001
		0.25 mM	1.75 mM	0.0021±0.0001	0.0011±0.0003	0.0012±0.0002
		0.50 mM	1.50 mM	0.0020±0.0001	0.0019±0.0006	0.0008±0.0002
		0.75 mM	1.25 mM	0.0018±0.0001	0.0015±0.0004	0.0008±0.0001
		1.00 mM	1.00 mM	0.0017±0.0001	0.0018±0.0005	0.0012±0.0002
		1.25 mM	0.75 mM	0.0016±0.0001	0.0019±0.0004	0.0014±0.0003
		1.50 mM	0.50 mM	0.0016±0.0001	0.0023±0.0006	0.0014±0.0003
		1.75 mM	0.25 mM	0.0015±0.0001	0.0036±0.0009	0.0025±0.0006

Nomenclature is as follows: BcSTFs, bovine cardiac synthetic thick filaments; BcS1, bovine cardiac myosin sub-fragment S1; BcHMM, bovine cardiac heavy meromyosin; In each experiment, the total concentration of the nucleotide (ADP+ATP) is 2 mM. Data are reported as mean±SEM ($n \geq 8$ from two experiments for each group). All myosin samples used here were not subjected to dephosphorylation.

Table S3. Parameters derived using single ATP turnover experiments in various myosin groups in response to various experimental factors

Effect of myosin RLC phosphorylation on single mant-ATP turnover kinetic parameters derived in porcine WT STFs

Parameter	[KCl]	PcSTF-Phos	PcSTF-Dephos
SRX population, A_{slow} (%)	30 mM	25.9±0.77	45.7±3.7
DRX ATPase rate, k_{fast} (s^{-1})	30 mM	0.012±0.001	0.020±0.002
SRX ATPase rate, k_{slow} (s^{-1})	30 mM	0.002±0.0001	0.004±0.0004

Effect of KCl on single mant-ATP turnover kinetic parameters derived in porcine WT and R403Q STFs

Parameter	[KCl]	PcSTF-WT	PcSTF-R403Q
SRX population, A_{slow} (%)	30 mM<br>100 mM	45.7±3.7<br>13.5±2.3	25.7±2.5<br>16.8±3.2
DRX ATPase rate, k_{fast} (s^{-1})	30 mM<br>100 mM	0.020±0.002<br>0.013±0.001	0.020±0.001<br>0.014±0.001
SRX ATPase rate, k_{slow} (s^{-1})	30 mM<br>100 mM	0.004±0.0004<br>0.003±0.0007	0.003±0.0002<br>0.002±0.0002

Effect of ADP on single mant-ATP turnover kinetic parameters derived in porcine WT and R403Q STFs

Parameter	[KCl]	Nucleotide chase	PcSTF-WT	PcSTF-R403Q
SRX population, A_{slow} (%)	30 mM	ATP chase<br>ADP chase	45.7±3.7<br>17.8±1.7	25.7±2.5<br>17.7±1.1
DRX ATPase rate, k_{fast} (s^{-1})	30 mM	ATP chase<br>ADP chase	0.02±0.002<br>0.035±0.005	0.02±0.001<br>0.018±0.001
SRX ATPase rate, k_{slow} (s^{-1})	30 mM	ATP chase<br>ADP chase	0.005±0.0004<br>0.003±0.0003	0.003±0.0003<br>0.0015±0.0003

Effect of ADP on single mant-ATP turnover kinetic parameters derived in different bovine WT cardiac systems

Parameter	[KCl]	Nucleotide chase	BcSTFs	BcHMM	BcS1
SRX population, A_{slow} (%)	30 mM	ATP chase<br>ADP chase	17.7±1.0<br>9.5±0.3	7.2±0.2<br>9.9±0.8	20.2±3.5<br>17.2±2.7
DRX ATPase rate, k_{fast} (s^{-1})	30 mM	ATP chase<br>ADP chase	0.013±0.0006<br>0.017±0.0004	0.010±0.0001<br>0.011±0.0002	0.014±0.0008<br>0.014±0.0007
SRX ATPase rate, k_{slow} (s^{-1})	30 mM	ATP chase<br>ADP chase	0.0022±0.0002<br>0.0016±0.0001	0.0014±0.0001<br>0.0017±0.0001	0.0020±0.0003<br>0.0014±0.0002

Nomenclature is as follows: BcSTFs and PcSTFs refer to bovine and porcine cardiac synthetic thick filaments, respectively; BcS1, bovine cardiac myosin sub-fragment S1; BcHMM, bovine cardiac heavy meromyosin; Phos, phosphorylated myosin; Dephos, dephosphorylated myosin, WT, wild-type. Data are reported as mean±SEM (n≥4 from two experiments for each group). All myosin samples used here, except those used for the 'Effect of myosin RLC phosphorylation' experiments, were not subjected to dephosphorylation.

Table S4. Potencies (EC_{50}/IC_{50} in μM) of mavacamten for different parameters derived using single ATP turnover experiments in healthy and diseased cardiac systems

Effect of mavacamten in different WT bovine cardiac systems

System	Factors	SRX population, (A_{slow})	DRX ATPase, (k_{fast})
BcSTFs	WT	0.66±0.05	0.31±0.05
BcHMM	WT	1.17±0.13	0.54±0.08
BcS1	WT	2.16±0.19	0.67±0.09

Effect of mavacamten in different healthy and disease STFs

System	Factors	EC_{50} (in μM) for SRX population, (A_{slow})	IC_{50} (in μM) for DRX ATPase, (k_{fast})
BcSTFs	ATP	0.63±0.06	0.41±0.06
	ADP	>4.35±0.64	1.14±0.20
BcSTFs	α-cardiac	1.83±0.25	0.61±0.08
	β-cardiac	1.03 ±0.11	0.35±0.06
PcSTFs	WT	1.76±0.22	0.29±0.02
	R403Q	2.00±0.28	0.30±0.02

Nomenclature is as follows: BcSTFs and PcSTFs refer to bovine and porcine cardiac synthetic thick filaments, respectively; BcS1, bovine cardiac myosin sub-fragment S1; BcHMM, bovine cardiac heavy meromyosin; α-cardiac, α-cardiac full-length myosin from the left atria; β-cardiac, β-cardiac full-length myosin from the left ventricle, WT, wild-type; EC_{50}/IC_{50} are the concentrations of mavacamten required to attain the half-maximal increase/decrease in a given parameter, respectively. Data are reported as mean±SEM ($n \geq 4$ from two experiments for each group). All myosin samples used here were not subjected to dephosphorylation.

SUPPLEMENTAL FIGURES

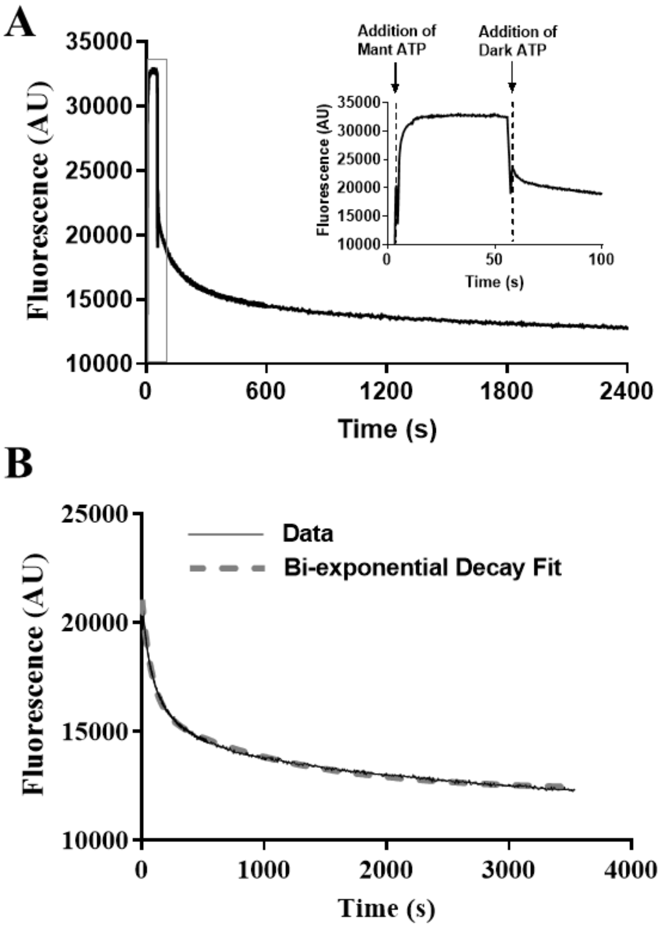

Figure S1. Representative trace in bovine cardiac synthetic thick filaments showing the timing of events in the single mant-ATP turnover experiment. (**A**) Time course of fluorescence showing mant-ATP binding followed by the dissociation of mant-nucleotides during the chase with non-fluorescent ATP. The inset of panel A shows an expanded view of the portion highlighted in the main panel, showing the time points at which mant-ATP and non-fluorescent ATP were added. (**B**) Full-time course (60 minutes) of the decay phase during the chase of mant-nucleotides with non-fluorescent ATP. The decay profile characteristically depicts two phases, a fast phase followed by a slow phase. Four different parameters (A_{fast}, k_{fast}, A_{slow}, and k_{slow}) were derived by fitting each trace to a bi-exponential decay function, where A represents the % amplitude, and k represents the observed ATP turnover rate of each phase. The dotted line in panel B represents the bi-exponential fit to the original trace (in black) with an r^2 value of 0.99. The values of A_{fast}, k_{fast}, A_{slow}, and k_{slow} in this representative trace are 72.6%, 0.012 s^{-1}, 27.4%, and 0.001 s^{-1}, respectively.

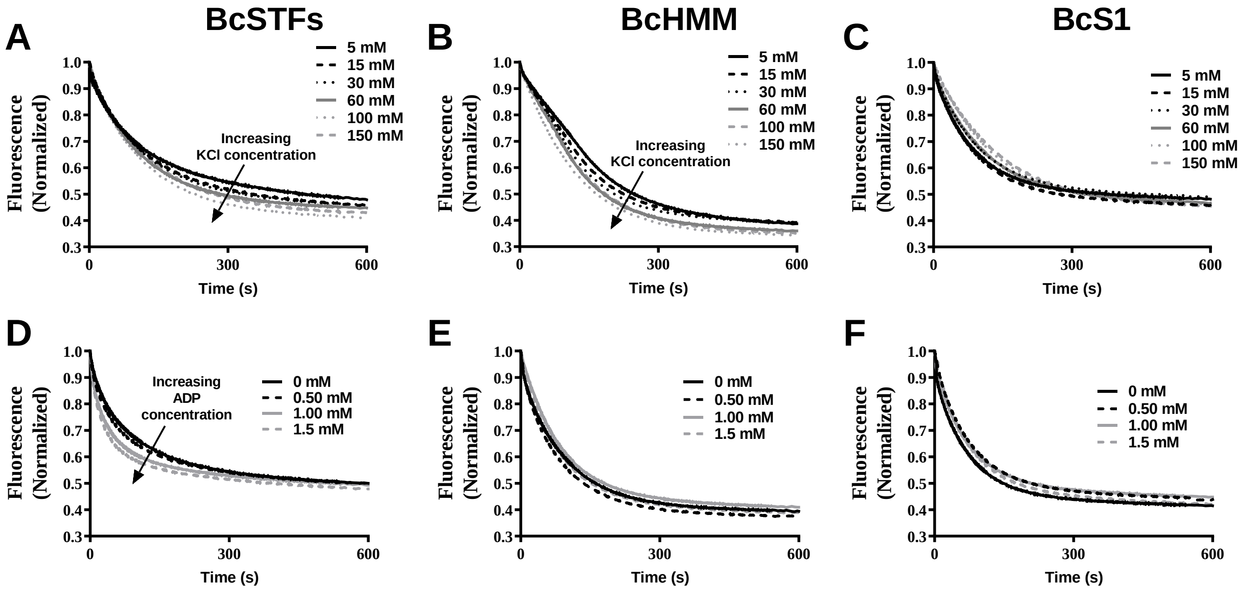

Figure S2. Effect of increasing KCl and ADP concentrations on the fluorescence decay profile in single mant-ATP turnover experiments. Comparing the effect of increasing KCl concentration on the fluorescence decay profiles during the chase phase in (**A**) bovine cardiac synthetic thick filaments (BcSTFs), (**B**) bovine cardiac heavy meromyosin (BcHMM), and (**C**) bovine cardiac myosin subfragment-1 (BcS1), respectively. Increasing the KCl concentration causes a leftward shift in the fluorescence decay profiles in both BcSTFs and BcHMM, suggesting that a greater fraction of mant-nucleotides dissociate from myosin during the fast phase (marked as black arrows in panels A and B). However, this phenomenon was not observed in BcS1. Comparing the effect of increasing ADP concentration on the fluorescence decay profiles during the chase phase in (**D**) BcSTFs, (**E**) BcHMM, and (**F**) BcS1, respectively. Increasing the ADP concentration leads to a leftward shift in the fluorescence decay profiles measured in BcSTFs (marked as a black arrow in panel D). However, this phenomenon was not observed in either BcHMM or BcS1. Four different parameters (A_{fast}, k_{fast}, A_{slow}, and k_{slow}) were derived by fitting each trace to a bi-exponential decay function with r^2 values ranging from 0.95 to 0.99, where A represents the % amplitude, and *k* represents the observed ATP turnover rate of each phase. In all panels, data were normalized to the fluorescence value at the onset of the decay.

A

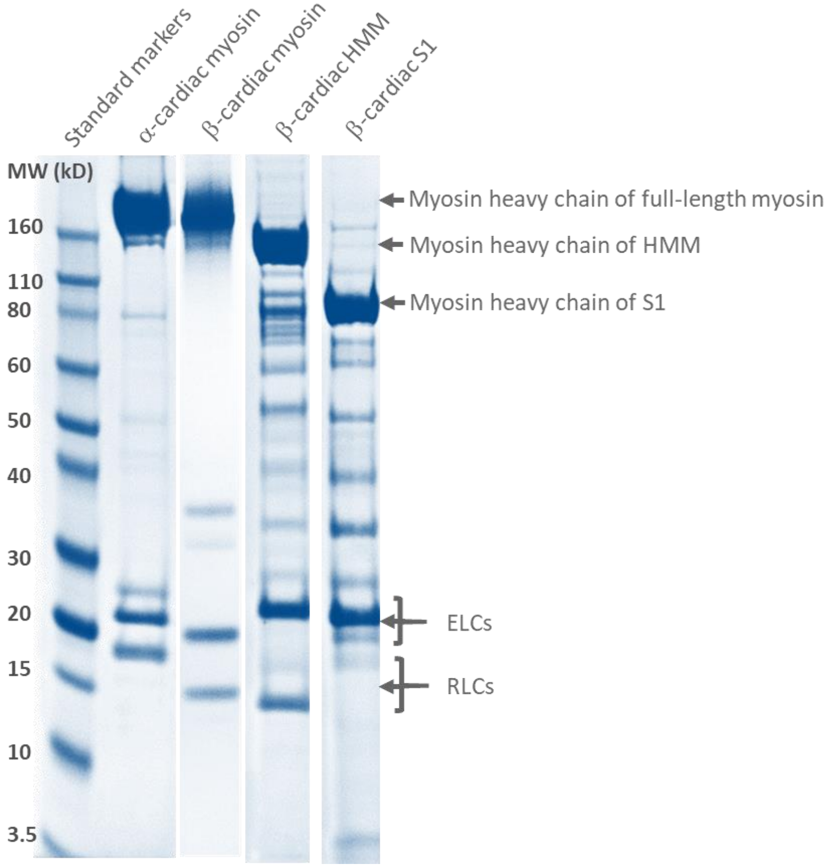

B

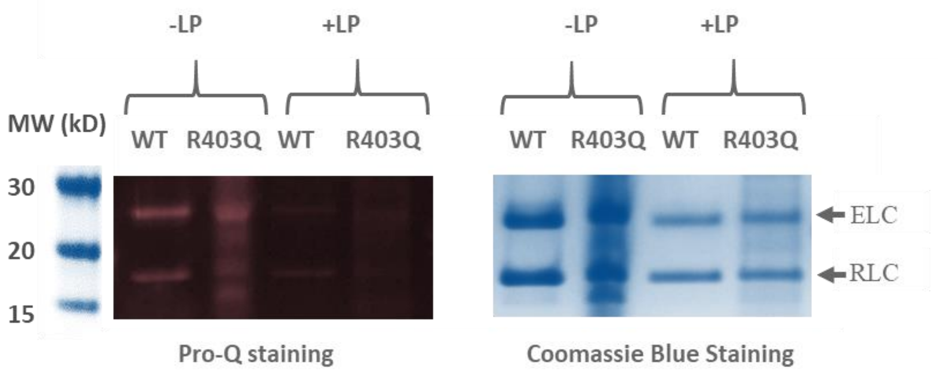

Figure S3. SDS-PAGE analysis. (**A**) An overloaded Coomassie blue-stained SDS-PAGE image of different myosin systems. The heavy chains and light chains are marked appropriately. (**B**) SDS-gel analysis of the phosphorylated proteins before and after treatment with lambda phosphatase (LP). The left panel is an image of Pro-Q stained gel showing the phosphorylation status of proteins in WT and R403Q myosin samples before and after treatment with LP, while the right panel shows the total amount of respective proteins following Coomassie blue staining. The phosphorylation levels of RLC and ELC in wild-type and R403Q myosin samples substantially decreased following treatment with LP.

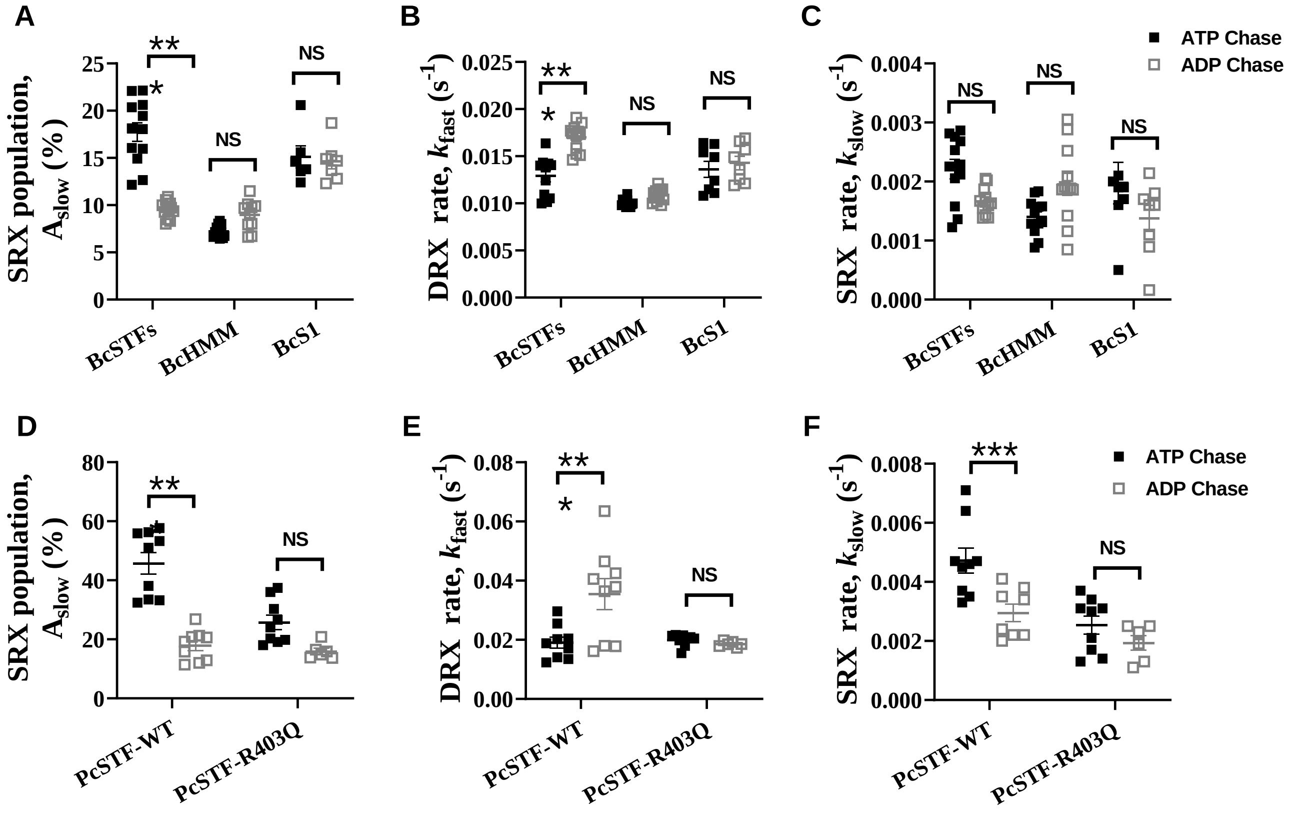

Figure S4. Effect of ATP chase versus ADP chase on the parameters derived from single ATP turnover experiments in various myosin systems. Panels A, B, and C show comparisons between BcSTFs, BcHMM, and BcS1 regarding the effect of ADP versus ATP chase on A_{slow}, k_{fast}, and k_{slow}, respectively. BcSTFs refers to the bovine cardiac synthetic thick filaments, BcS1 refers to bovine myosin subfragment-1, and BcHMM refers to bovine cardiac heavy meromyosin. Panels D, E, and F show comparisons between PcSTF-WT and PcSTF-R403Q regarding the effect of ADP versus ATP chase on A_{slow}, k_{fast}, and k_{slow}, respectively. PcSTF-WT and PcSTF-R403Q refer to porcine cardiac synthetic thick filaments made of WT and mutant R403Q myosin, respectively. Statistical differences presented for bovine systems were based on one-way ANOVA and subsequent post-hoc comparisons, while those for porcine systems were based on a two-tailed *t*-test (***P<0.001; NS, not significant). Data are expressed as mean±SEM ($n \geq 6$ for each).

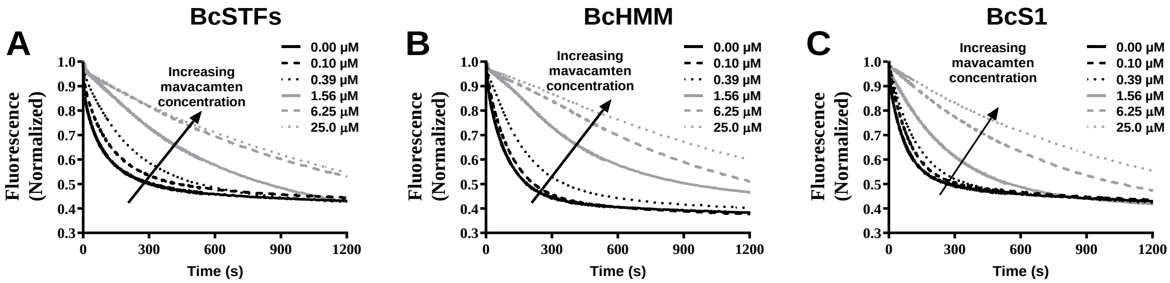

Figure S5. Effect of increasing mavacamten concentrations on the fluorescence decay profile in single mant-ATP turnover experiments. Representative comparisons showing the effect of increasing concentrations of mavacamten on the fluorescence decay profiles in (**A**) bovine cardiac synthetic thick filaments (BcSTFs), (**B**) bovine cardiac heavy meromyosin (BcHMM), and (**C**) bovine cardiac myosin subfragment-1 (BcS1). Increasing the concentration of mavacamten leads to a progressive rightward shift in the fluorescence decay profiles, suggesting a slower release of mant-nucleotides from myosin regardless of the myosin system used. In all panels, data were normalized to the fluorescence value at the onset of the decay. Four different parameters (A_{fast}, k_{fast}, A_{slow}, and k_{slow}) were derived by fitting each trace to a bi-exponential decay function with r^2 values ranging from 0.95 to 0.99, where A represents the % amplitude, and *k* represents the observed ATP turnover rate of each phase.

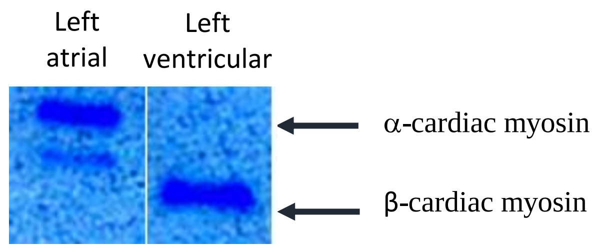

Figure S6. Native-PAGE analysis. Native PAGE gel showing the distribution of myosin isoforms in bovine left atrial and bovine left ventricular muscles. As shown, bovine left atrial tissue predominantly expresses an α-cardiac myosin isoform that migrates slower on the gel. In contrast, bovine left ventricular tissue predominantly expresses β-myosin isoform that migrates faster on the gel.
